# Research Progress on Interfacial Design and Mechanical Optimization of Graphene-Reinforced Titanium Matrix Composites

**DOI:** 10.3390/ma19040822

**Published:** 2026-02-21

**Authors:** Yongkang Fu, Shilong Xing, Zongan Li, Shuo Wu, Liran Sun, Xiaohua Yang, Wei Shen, Zhikun Li, Xiaocong Li

**Affiliations:** 1 School of Navigation and Shipping, Shandong Jiaotong University, Weihai 264209, China; 2 School of Construction Machinery, Shandong Jiaotong University, Jinan 250357, China

**Keywords:** graphene; titanium matrix composites; mechanical properties, dispersion control, interface reaction

## Abstract

Graphene (GR) demonstrates significant potential in enhancing the mechanical performance of titanium matrix composites (TMCs), particularly by improving their tensile strength, fracture toughness, and fatigue resistance, thereby optimizing the overall structural integrity and durability of the composites; however, their practical implementation confronts two fundamental challenges: achieving uniform dispersion and mitigating excessive interfacial TiC formation, which compromises mechanical properties. This review comprehensively explores progress in the fabrication, interfacial design, and mechanical optimization of TMCs reinforced with graphene-based materials. Various processing techniques, such as powder metallurgy (PM) and spark plasma sintering (SPS), are critically analyzed in terms of their advantages and limitations for producing high-performance TMCs. This article analyzes how key parameters in processes like PM and SPS affect graphene structure, dispersion, and interfacial reactions. It outlines strategies—including surface modification, 3D structural design, and multiscale interface engineering—that enhance both strength and toughness. While progress has been made in microscale performance, challenges remain in engineering stability and long-term reliability. Future work should focus on intelligent process optimization and architectured composite manufacturing. By systematically synthesizing existing research findings, this article clarifies the advantages and limitations of current technological approaches, providing a theoretical foundation and technical roadmap for the subsequent development of graphene-reinforced TMCs that exhibit high strength, high toughness, and excellent reliability.

## 1. Introduction

High strength and low density are objectives continuously pursued in industrial fields such as aviation, aerospace, and marine vessels. Titanium (Ti) and its alloys are renowned for their exceptional strength-to-weight ratio, enabling a substantial reduction in material usage without compromising structural integrity. This results in lighter equipment, making them ideal for a wide range of engineering and industrial applications. Their high specific strength, excellent resistance to high temperatures and corrosion, and compatibility with composite materials further enhance their versatility in demanding environments [1,2,3,4]. With the advancement of aerospace, marine, and related fields, titanium alloys are required to exhibit higher performance in terms of high-temperature resistance, corrosion resistance, and creep resistance. As a result, titanium or titanium alloys alone may be inadequate to meet the demanding mechanical properties required for critical components in these applications [5]. Researchers have made many attempts to develop higher-performance titanium matrix composites (TMCs). Second-phase strengthening is a widely employed and viable approach to significantly improve the performance of titanium-based materials. Graphene (GR) has been identified as a promising reinforcement, significantly enhancing the mechanical performance of TMCs [6,7]. GR, a widely utilized nanocarbon material, is characterized by a Young’s modulus of approximately 1 TPa and a theoretical strength of around 130 GPa [8,9], but its two-dimensional layered structure also effectively impedes dislocation motion, enhancing both the strength and toughness of the Ti matrix. Its intrinsic flexibility further contributes to improved composite plasticity [10]. More importantly, compared to one-dimensional nanomaterials like carbon nanotubes (CNTs), the planar geometry of graphene provides a larger interfacial contact area for more efficient load transfer; its two-dimensional morphology also shows greater potential in crack inhibition and constructing three-dimensional reinforcement networks, typically offering better cost-effectiveness and process compatibility [11,12]. The two-dimensional layered structure of graphene is subdivided into different professional terms based on its specific morphology and number of layers. The most prominent types of graphene described in this text include Graphene Nanoplatelets (GNPs), Graphene Nanoflakes (GNFs), Graphene Nanosheets (GNSs), multi-layer graphene (MLG), and others. These all represent specific manifestations of the two-dimensional layered graphene structure at different scales and states. They are briefly introduced in Appendix A.

However, the reinforcing efficiency of graphene in the titanium matrix is not determined solely by its intrinsic properties but critically depends on several specific characteristics: its structural integrity (defect density), number of layers and lateral size, as well as surface chemical state. These characteristics collectively govern its dispersion behavior, the extent of interfacial reactions, and the ultimate load transfer efficiency within the composite. Therefore, selecting and preserving graphene with low defect density, appropriate dimensions, and a controlled surface state constitutes a fundamental prerequisite for realizing its reinforcing potential.

**Figure 1:**
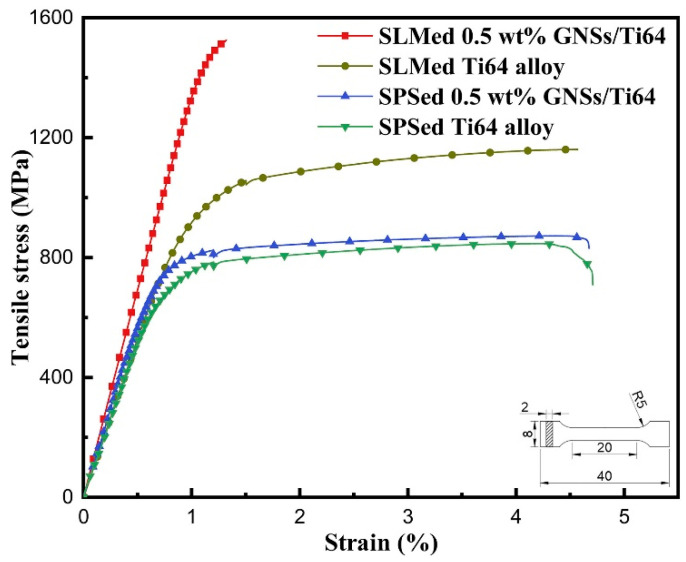
Stress–strain curves of 0.5 wt.% GNSs/Ti64 and Ti64 alloys [13].

To fabricate high-performance graphene-reinforced TMCs, a variety of preparation routes have been developed and explored. Yan et al. [13] used Ti-6Al-4V (Ti64) as the matrix material and GNSs as the reinforcement. As shown in Figure 1, both processing technologies highlight the enhanced mechanical properties of Ti64 achieved by the addition of GNSs. In particular, the 0.5 wt.% GNSs/Ti64 composite fabricated via SLM exhibits a tensile strength of 1517 MPa, albeit at the cost of some ductility. Mutuk et al. [14] fabricated dual-reinforced TMCs via powder metallurgy using a binary powder mixture of silicon nitride (Si_3_N_4_) and GNPs. When the addition amounts of GNPs and silicon nitride are 0.15 wt.% and 3 wt.% respectively, the composite exhibits optimal performance, with peak hardness and compressive strength reaching 634 HV and 1458 MPa, respectively, indicating a substantial improvement in mechanical performance. These findings confirm the effectiveness of graphene-based reinforcements in enhancing the properties of TMCs [15,16,17].

Current research in this field has shown significant advances in optimizing mechanical properties and refining microstructures. However, achieving uniform dispersion of graphene and precisely controlling the interfacial reactions between graphene and the Ti matrix remain two core challenges. This review aims to systematically analyze the microstructural evolution and strengthening mechanisms associated with various processing routes, assess the capabilities and limitations of existing strategies for enhancing mechanical performance, and outline promising research directions to overcome the current challenges.

## 2. Fabrication Methods of Graphene-Reinforced Titanium Matrix Composites

Selecting optimal fabrication methods and processing parameters for uniform graphene dispersion and enhanced interfacial bonding remains a critical challenge, especially for TMCs due to titanium’s high reactivity and graphene’s strong van der Waals forces, which lead to agglomeration and brittleness. The manufacturing technologies for TMCs have become diverse, but significant differences exist in microstructural control, reinforcement distribution, and material performance. Powder metallurgy, due to its compatibility with titanium, has become the preferred technology for producing high-performance TMCs. This section reviews powder metallurgy and spark plasma sintering, analyzing the advantages and limitations of these methods.

### 2.1. Powder Metallurgy

Powder metallurgy is a key technique for fabricating graphene-reinforced TMCs. Its core procedures encompass uniform mixing of titanium powder with graphene, forming, and sintering. This technique enables near-net shaping, significantly reducing subsequent machining processes and material waste while achieving material utilization rates exceeding 90%. By adjusting powder composition, particle size, and processing parameters, material properties can be precisely tailored to meet specific performance requirements for composites. Furthermore, powder metallurgy effectively eliminates potential component segregation inherent in molten-state processing, ensuring homogeneous composition and consequently delivering enhanced and consistent performance [18,19].

**Figure 2:**
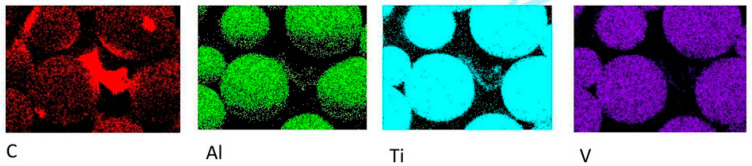
Area mapping image of mixture powder [21].

In powder metallurgy for TMCs, mechanical alloying (MA) introduces reinforcement phases, achieves microstructural homogenization, and enables alloying and grain refinement through high-energy deformation. Ball milling refines particle size, embeds reinforcements, and promotes atomic-level mixing. These mechanisms ultimately yield composite powders with homogeneous microstructures and controlled particle size distribution [20]. From a process design perspective, MA is regarded not only as a mixing step but also as an activation step for defect management. Its outcome should be constrained by measurable defects to achieve sufficient interface activation for embedding and atomic-level mixing. Sharma [21] uniformly mixed multi-layer graphene (MLG) with Ti64 powder for 8 h in a ball mill at a 5:1 ball-to-powder ratio (BPR). Homogeneous dispersion of MLG within the Ti64 matrix was confirmed via energy-dispersive spectroscopy mapping, as illustrated in Figure 2. This provided additional evidence that MA was an effective means of resolving GNP dispersion. The resulting composite containing 1.2 wt.% MLG exhibited a hardness of 5.29 GPa, representing a 68.4% increase compared to unreinforced Ti64 (3.14 GPa).

**Figure 3:**
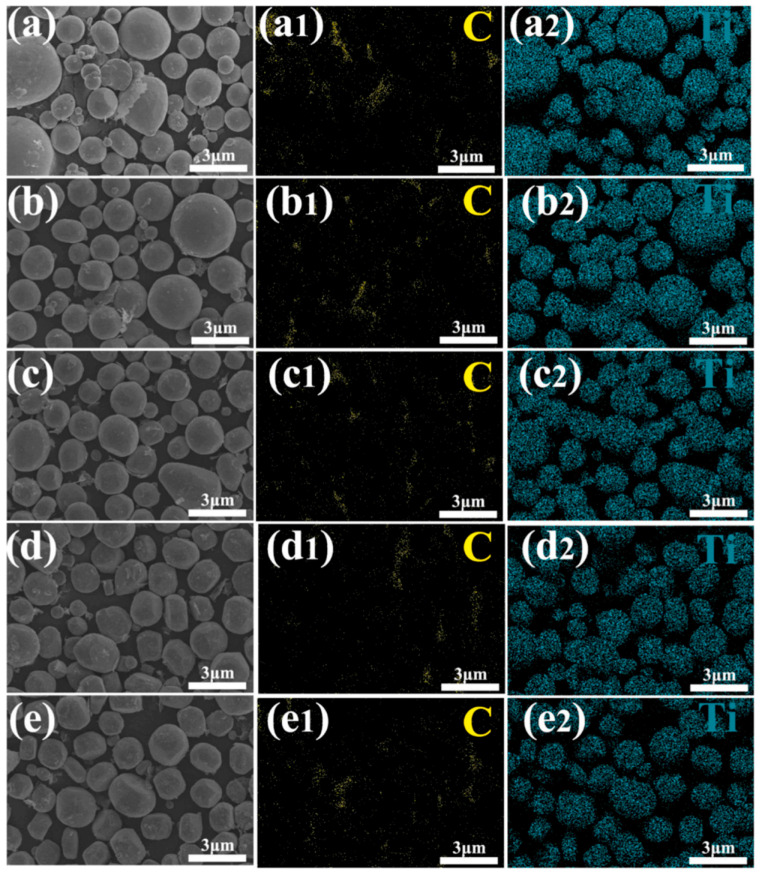
SEM images of TiB2@GNPs/TC4 ball-milling powder with different milling time: (**a**) 2 h, (**b**) 4 h, (**c**) 8 h, (**d**) 16 h, (**e**) 24 h; the corresponding EDX analysis of C and Ti: (**a1**–**e1**), and (**a2**–**e2**), respectively [23].

**Figure 4:**
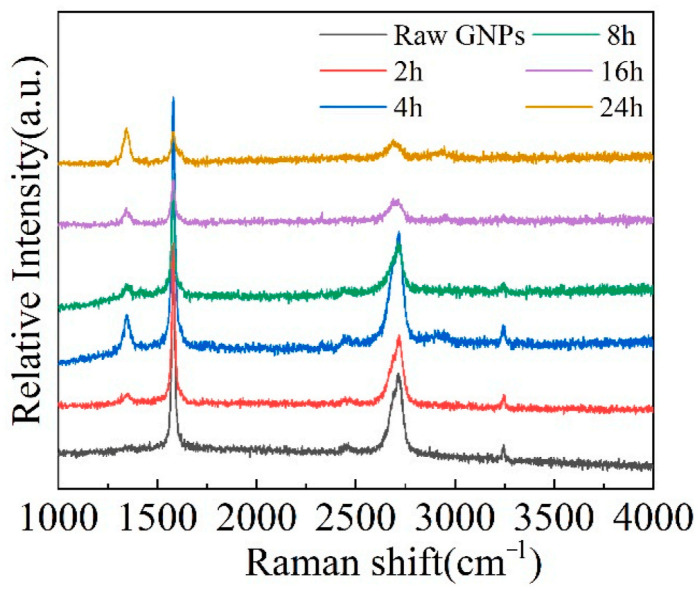
Raman spectrum of the mixed powders with various ball-milling times [23].

**Figure 5:**
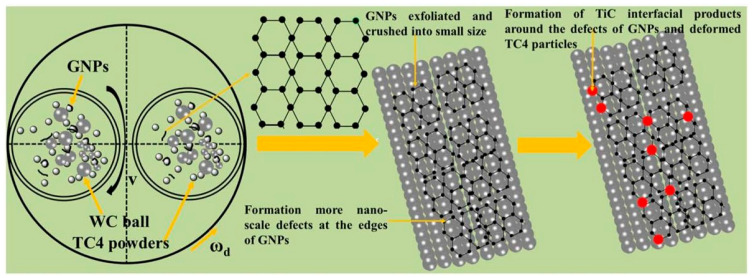
Schematic diagram of interfacial product TiC formation and evolution of GNPs during the ball-milling process [24].

Ball-milling parameters significantly affect the structural evolution and integrity of graphene. Insufficient milling may cause poor embedding and uneven distribution, leading to agglomeration and weak bonding. Prolonged milling improves distribution but degrades the graphene structure, reducing its reinforcing ability [22]. Wang et al. [23] ball-milled TC4 alloy powder, GNPs, and TiB_2_ particles in a planetary mill at varying durations (2, 4, 8, 16, 24 h) with a BPR of 5:1 and rotational speed of 250 rpm, using 20 wt.% anhydrous ethanol as the process control agent. TC4 particles remained spherical up to 8 h (Figure 3a–c), but prolonged milling flattened them into flake-like structures (Figure 3d,e), while GNP dispersion improved with longer milling (Figure 3a1–e1). However, Raman spectroscopy (Figure 4) revealed that as the ball-milling time increased from 2 h to 24 h, the progressive increase in the I_D_/I_G_ ratio correlated with a rise in the defect density within the GNPs. Notably, the TC4 powder milled for 8 h exhibited a defect level (I_D_/I_G_ = 0.51) and a graphene layer characteristic (I_2D_/I_G_ = 0.86) that remained within an acceptable range. This optimal condition achieved a high degree of dispersion while avoiding excessive structural damage to the GNPs. The optimal milling time for a given protocol is contingent on its specific parameters. Zhou et al. [24] also investigated the regulatory mechanisms of ball-milling duration on microstructural evolution and interfacial reactions. The results revealed that short-term milling (2–5 h) induced cold welding of TC4 particles, increasing their size while partially exfoliating graphene that adhered to particle surfaces. Conversely, prolonged milling (≥10 h) triggered TC4 fragmentation and elevated the I_D_/I_G_ ratio to 1.52 (Table 1), indicating severe structural degradation of GNPs leading to amorphization. Simultaneously, GR became embedded within the matrix and reacted with Ti to form nanoscale TiC phases. Excessive TiC formation progressively impaired the composite’s ductility, with Figure 5 delineating the nucleation and evolution of TiC. Consequently, the process design must balance dispersion quality with structural integrity. Insufficient ball milling leads to poor dispersion and weak interfaces, while excessive milling damages the graphene structure and promotes TiC formation, reducing the strengthening effect. The optimal window is narrow and process-dependent, requiring a balance between uniform embedding and strong bonding before GR degradation occurs.

Despite the confirmed ability of MA to improve GNP dispersion in the Ti matrix, prevailing research has predominantly centered on isolated performance outcomes or a singular ball-milling parameter, failing to capture the complexity of the multi-stage powder metallurgy process. Across distinct processing stages of powder metallurgy, the influence mechanisms of processing parameters on fabricated TMCs vary significantly. However, the synergistic effects among parameters across different stages remain insufficiently explored. A systematic understanding of these interactions is essential for optimizing the overall performance of TMCs. Table 2 systematically summarizes the core parameters governing TMC quality and their underlying mechanisms.

As an inorganic material, GNFs are characterized by strong Van der Waals forces—which promote agglomeration—and a significant density difference from Ti powder. These combined factors make homogeneous mixing via direct MA difficult to achieve. For improved GR dispersion, an effective solution involves depositing an electroless nickel (Ni) transition layer onto GNP reinforcement surfaces. Mu et al. [40] employed an electroless plating method to coat GNFs with Ni nanoparticles, followed by ball milling for dispersion. Compared to uncoated GNF/Ti composites, the resulting Ni-GNFs were distributed within the Ti matrix at sizes of 3–20 μm without significant agglomeration. This demonstrates that the Ni nanoparticle coating mitigates the van der Waals forces between GNFs, preventing agglomeration; simultaneously, it increases the density of GNFs, reducing the density mismatch with Ti powder; collectively, these effects facilitate uniform mixing. Research has further demonstrated that Ni serves a dual purpose: it not only weakens the van der Waals forces between GNFs, but its coatings also act as reaction barriers. At high temperatures, Ni preferentially reacts with the Ti substrate to form intermetallic compounds, thereby suppressing excessive TiC formation. Additionally, Ni promotes the formation of strongly bonded multi-layered interfaces [41]. Prior to ball milling, graphene can be subjected to ultrasonic pretreatment to achieve a stable dispersion, followed by milling with Ti powder. This process effectively disrupts the initial agglomerates and enhances dispersibility. However, caution is warranted because Ni–P coatings can precipitate brittle intermetallics at elevated temperatures and shift the Ti α/β balance; therefore, prioritize thin, uniform low-phosphorus or Ni–B coatings, limit total Ni input, and combine Spark Plasma Sintering (SPS) with rapid heating and short dwells to confine the Ti–Ni layer. Surface modification and physical dispersion techniques are complementary rather than mutually exclusive—their combination can synergistically address dispersion and interface challenges, especially for high-graphene-content composites. Moreover, the choice of modification/dispersion strategy depends on performance trade-offs: chemical modification optimizes both dispersion and interfacial bonding but risks introducing impurities, while physical methods preserve graphene’s intrinsic structure yet may require post-processing to enhance interface adhesion.

**Figure 6:**
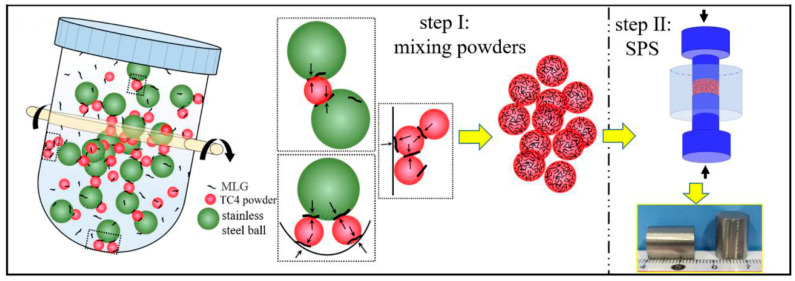
Schematic illustration of fabricating MLG/TC4 composites, including step I (3D mixing of powders) and step II (SPS) [43].

Powder metallurgy (PM) addresses critical challenges in graphene-reinforced TMCs—such as weak interfacial bonding and the strength-ductility trade-off—through precise control of graphene dispersion, interfacial reaction pathways, and microstructure design. For instance, three-dimensional dynamic mixing and flake encapsulation techniques effectively mitigate agglomeration issues caused by the high specific surface area of graphene. In the 3D dynamic mixing process, titanium matrix powders and graphene with varying contents are sealed in a polytetrafluoroethylene jar together with stainless-steel balls. Under low-speed rotation, the system facilitates omnidirectional tumbling that generates mild compressive and shear stresses, enabling graphene to initially remain suspended within the container before achieving complete interfacial contact and adhesion with the titanium powder surfaces. The low-energy input of this process is critical—it disrupts graphene agglomerates via gentle mechanical action without inducing defects that compromise graphene’s intrinsic mechanical properties. Figure 6 illustrates the 3D dynamic mixing process of MLG/TC4 composites. Without the need for surface modification, this method simplifies the processing route and effectively resolves the agglomeration problems commonly associated with conventional techniques—such as structural damage to graphene caused by high-energy ball milling or the requirement for additional post-treatment after ultrasonic dispersion. This approach lays a solid foundation for the subsequent formation of a three-dimensional network structure [42,43].

**Figure 7:**
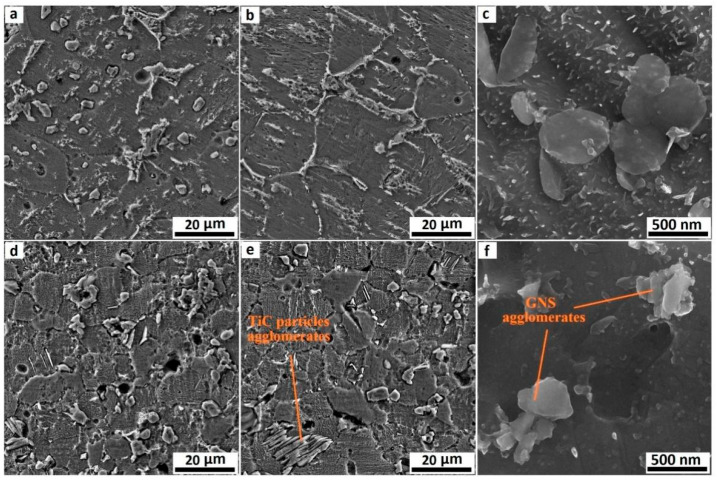
SEM image of Ti/GNS composite containing 1 and 1.5 wt.% of GNSs, (**a**) Ti/1GNS composite sintered for 1 h, (**b**,**c**) Ti/1GNSs composite sintered for 5 h, (**d**) Ti/1.5GNSs composite sintered for 1 h, and (**e**,**f**) Ti/1.5GNSs composite sintered for 5 h [25].

During the subsequent sintering/densification stage in PM, particularly during SPS or high-temperature heat treatment, titanium readily reacts with graphene to form TiC. This in situ-formed TiC significantly enhances chemical bonding and load-transfer efficiency at the graphene-matrix interface, thereby improving yield strength and hardness. However, excessive interfacial reactions produce counterproductive effects, diminishing the intrinsic strengthening mechanism [39,44]. From a materials design perspective, achieving an optimal balance between interfacial chemical bonding and the preservation of graphene’s intrinsic properties remains a critical scientific challenge. Herein, the PM process demonstrates its advantage by enabling precise control over the degree of in situ reaction between graphene and the titanium matrix through low-temperature, high-pressure, and rapid sintering. This controlled approach prevents excessive formation of brittle TiC and consequent interfacial embrittlement, ultimately resulting in a heterostructured synergistic interface characterized by residual graphene and nano-TiC [45]. Haghighi et al. [25] systematically investigated the effects of GNS content and sintering parameters on TiC formation, microstructure, and mechanical properties. As shown in Figure 7a–c, with the sintering time extending from 1 h to 5 h, the distribution of TiC particles within the titanium matrix becomes more uniform, accompanied by a significant reduction in pore concentration. This trend aligns with the principle that prolonged sintering enhances atomic diffusion and improves densification. When the GNS content increases (Figure 7e,f), the excessive GNS acts as an additional carbon source for TiC formation, leading to a substantial increase in the volume fraction of TiC particles. However, these TiC particles no longer maintain a homogeneous dispersion; instead, they form noticeable agglomerates preferentially located at the matrix grain boundaries. Such grain-boundary segregation of TiC breaks the continuous load transfer path between the matrix and reinforcement, as the brittle TiC agglomerates are prone to cracking under external stress, triggering premature failure of the composite. This agglomeration disrupts the continuity of the titanium matrix and significantly degrades the uniformity of TiC distribution. On the other hand, the high specific surface area of GNS promotes agglomeration through weak interlayer van der Waals interactions. These agglomerates grow in size with extended sintering time and tend to initiate pore formation at the GNS-matrix interface, as indicated by the arrows in Figure 7f. Consequently, suppressing the initial agglomeration of GNS is as crucial as controlling the sintering kinetics to mitigate defect formation and realize the full potential of the composite.

PM, spanning from powder preparation to forming to sintering, inherently possesses the capability to simultaneously control reinforcement distribution, interface structure, and matrix organization across the nano-, sub-micro-, and microscale levels. This hierarchical control establishes a fundamental processing basis for multi-mechanism synergies.

At the nanoscale, the PM process enables a uniform coating of graphene onto titanium powder surfaces via mechanical alloying, while simultaneously introducing numerous lattice defects and high-energy interfaces during ball milling. In subsequent sintering stages, a controlled chemical reaction occurs between titanium and graphene, generating nano-sized TiC particles. This unique composite interface structure of partially retained graphene and in situ-formed TiC significantly enhances interfacial bonding strength and load transfer efficiency. Research demonstrates that through precise control of ball-milling energy and SPS parameters, an optimal balance can be achieved between maintaining graphene sheet integrity and promoting appropriate in situ phase reactions. This balance enables synergistic interaction between nanoscale load-transfer strengthening and interfacial phase strengthening, resulting in enhanced strength without excessive degradation of graphene’s intrinsic structure [46,47].

At the submicron scale, the high-energy deformation induced by mechanical alloying introduces a high density of dislocations, subgrain boundaries, and substructural defects within titanium powder. These defects are partially retained after densification, thereby serving as potent sources for dislocation strengthening and grain refinement [48]. The presence of GR further elevates the dislocation density, as its rigid phase interfaces impede dislocation motion, thereby promoting strain hardening.

**Figure 8:**
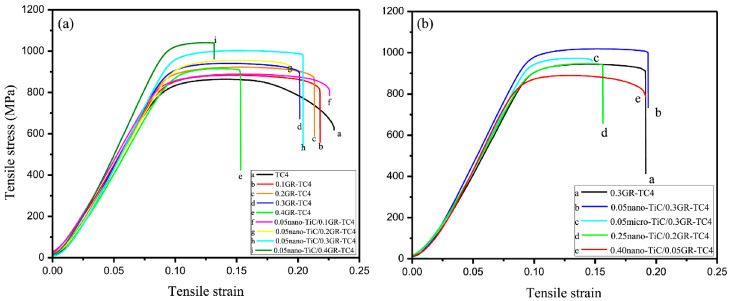
Tensile stress–strain curves of the TC4, GR-TC4 and nano-TiC/GR-TC4 composites (**a**); tensile curves of the GR-TC4, nano-TiC/GR-TC4, micro-TiC/GR-TC4 composites (**b**) [42].

At the microscale, PM enables precise control over the spatial distribution of reinforcements through graded powder mixing and subsequent thermomechanical deformation, allowing the construction of either network or layered architectures to achieve macroscopic homogenization of load and strain distribution. Additionally, microscale architecture design must synergize with the Ti matrix’s α/β phase characteristics. Under external loading, the networked TiC/GNP structure forms a continuous strengthening framework that facilitates efficient load transfer and impedes crack propagation, while coarser grain regions accommodate plastic deformation, thereby enhancing overall ductility. The bimodal grain structure—characterized by the coexistence of fine-grained strengthening zones and coarse-grained ductile regions—further provides an effective approach to balancing strength and plasticity [49]. Zhang [42] constructed a typical example of this architecture through 3D dynamic mixing combined with SPS, producing a GR-TiC hybrid-reinforced 3D network structure. As shown in Figure 8a, at the same GR content, the yield strength of nano-TiC/GR-TC4 is significantly higher than that of GR-TC4. This was attributed to the synergistic enhancement of GR/nano-submicron TiC through network structure by increasing the energy required for microcrack propagation and deflection. Figure 8b reveals that micro-sized TiC tends to cause stress concentration at interfaces, facilitating crack initiation, whereas nano-TiC disperses more uniformly, achieving a better balance between strength and ductility. The paradigm-shifting advantage of PM in synthesizing graphene-reinforced TMCs is its unparalleled capacity for multiscale collaborative design. This approach transcends conventional reinforcement strategies by synergistically integrating interface engineering at the nanoscale, defect architecture at the submicron scale, and spatial configuration at the microscale.

When the stress concentration around the reinforcements exceeds a critical value, interfacial debonding occurs between the reinforcement and the titanium matrix due to their plastic mismatch. These debonded interfaces then act as crack initiation sites, with the nascent cracks subsequently propagating into micro-cracks under continued loading. According to the crack propagation energy absorption equation given by Equation (1) [42]:

(1) $$Q=AL\sqrt{\frac{\pi EG}{{2(1-v)}^{2}}}\cdot d^{\frac{3}{2}}$$

**Figure 9:**
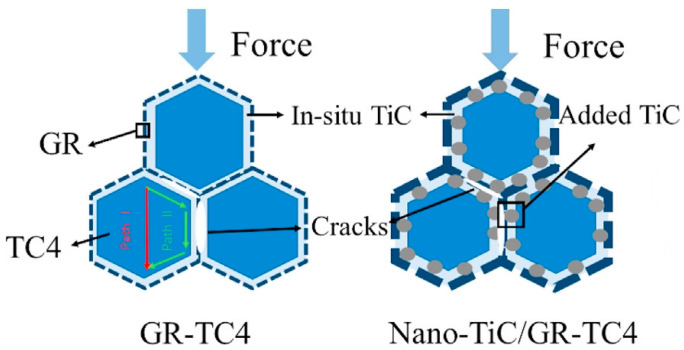
Schematic diagram of the network interface strengthening mechanism [42].

The variables are defined as follows: *E*, elastic modulus of reinforcements (Pa); *G*, critical strain energy release rate for dynamic crack propagation into the softer reinforcement phase (J·m^−2^); *ν*, Poisson’s ratio (dimensionless); d, reinforcement size along the crack direction (in meters; values reported in μm were converted to m for calculation). Parameters *L* and *A*, related to crack length and surface area, respectively (typically in m and m^2^), ensure that the resulting *Q* is in Joules. For the GR-TC4 composite, the plasticity mismatch between graphene and the matrix readily induces interfacial stress concentration, which subsequently triggers interfacial debonding and initiates crack formation. In contrast, within the nano-TiC/GR-TC4 composite, the presence of nano-TiC divides the crack propagation into two distinct paths: either through the matrix grains, resulting in transgranular fracture (Path I), or along the network boundary (Path II) [50]. When the crack propagates along path II (Figure 9), significantly more energy (*Q*) is absorbed, as indicated by Equation (1), where the values of *d* (reinforcement size in the crack direction) and *E* (elastic modulus of reinforcements) are much larger for Ti composites with dual reinforcing phases and ternary sizes (nano to submicron). This suggests that introducing nano-TiC into the composites to modify the interface structure plays a crucial role in enhancing the mechanical properties.

The future development of PM will prioritize closed-loop control and cost-effectiveness. Efforts will focus on optimizing the compatibility between low-energy ball milling and rapid sintering, as well as developing graphene pre-dispersed masterbatches or slurries for industrial consistency. Establishing a process-parameter database supported by statistical process control will help stabilize quality and facilitate the transition from laboratory research to engineering applications. For interfacial reactions, conventional sintering equipment will be leveraged to regulate TiC content and morphology through tailored heating profiles and atmosphere control.

### 2.2. Spark Plasma Sintering

Sintering is a key step in the powder metallurgy process, the essence of which is the formation of a dense and integrated body through diffusion, recrystallization, and phase reactions among powder particles. SPS technology generates high temperatures directly between powder particles through the application of a pulsed direct current. In contrast to conventional sintering methods, SPS enables simultaneous consolidation and sintering at lower temperatures, shorter dwelling times, and higher heating rates, resulting in fully densified compacts with refined microstructures. Owing to these advantages, SPS has been widely adopted in the fabrication of graphene-reinforced TMCs [51,52].

**Figure 10:**
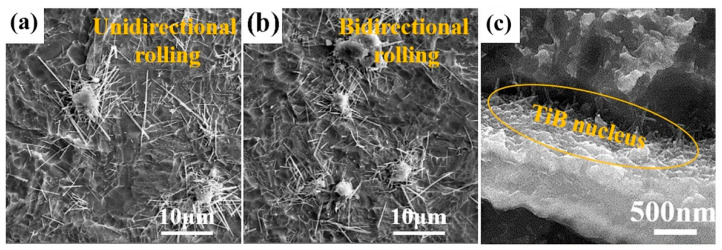
(**a**) TiB/Ti6A l4V composite prepared by unidirectional RHR, (**b**) TiB/Ti6Al4V composite prepared by bidirectional RHR, (**c**) TiB2/Ti6Al4V billet and local enlarged image [55].

Numerous studies have confirmed that SPS achieves rapid high densification of materials within remarkably short timeframes through Joule heating and plasma discharge effects generated by pulsed currents, while effectively suppressing grain growth. In practice, the densification advantage is best understood as the synergy of very high heating rates, concurrent uniaxial pressure, and short dwells. Falodun et al. [53] employed Ti6Al4V alloy powder as the matrix with 5–20 vol% SiAlON ceramic particles as additives. Through SPS at 1000–1100 °C under 35 MPa pressure for 10–30 min, the process leverages plasma activation effects and Joule heating generated by a pulsed current to enhance particle surface activity. This plasma activation not only promotes atomic diffusion but also inhibits SiAlON particle agglomeration. This approach achieved rapid densification, with relative densities exceeding 95%, within a short duration of 10–30 min. The combination of rapid heating rates (200 °C/min) and short sintering duration significantly suppresses grain coarsening, resulting in a fine-grained microstructure and a 58% increase in hardness. In a related study, Singh et al. [54] fabricated Ti6Al4V matrix composites via SPS, achieving >99% theoretical density in merely 5 min at 1200 °C. This rapid consolidation avoided the grain coarsening associated with prolonged high-temperature exposure in conventional sintering. Additionally, the high pressure (50 MPa) and electric-field-assisted densification during SPS reduced material porosity. Wang et al. [55] conducted SPS at 650 °C under 300 MPa pressure for 10 min to produce high-density TiB_2_/Ti6Al4V preforms. These preforms subsequently underwent bidirectional reaction hot rolling (RHR) at 950 °C for 8 passes, ultimately fabricating TiB/Ti6Al4V composites with excellent isotropy. Figure 10a,b display the microstructure of DRTMC after unidirectional and bidirectional RHR processing. The SPS-sintered preforms achieved a relative density of 98.6% ± 0.1%, with a partial reaction of TiB_2_ forming TiB nuclei at low sintering temperatures, shown in Figure 10c. Yu et al. [37] employed SPS technology to sinter GNP/TC21 composites across a temperature range of 850–1050 °C. They observed that when the sintering temperature increased from 850 °C to 1050 °C, the relative density of the composites rose from 94.57% to 99.35%. It is indicated that appropriately increasing the sintering temperature contributes to further promoting the densification process. However, it must be noted that any temperature increase must be constrained by the Ti-C reaction threshold. Excessively high temperatures will lead to the formation of substantial TiC. Therefore, the heating rate and soaking time during SPS must be strictly controlled.

**Figure 11:**
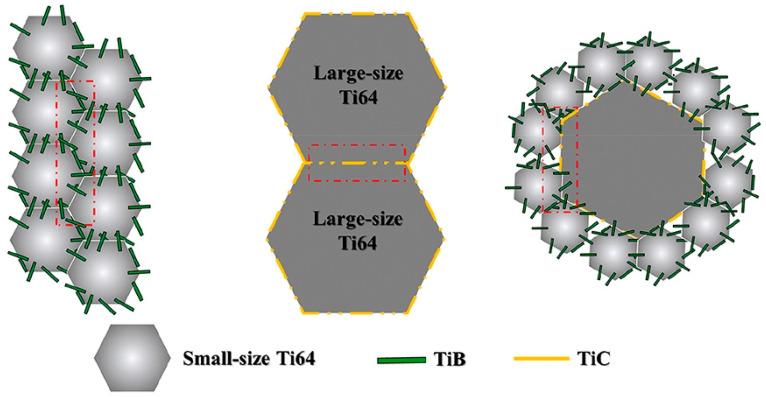
The schematic diagram illustrates the interface bonding patterns in the heterogeneous network structure (B + GOs)/Ti64 composites [56].

The characteristic extremely high heating rates and short dwell times of the SPS process effectively suppress grain-boundary diffusion and growth, resulting in a refined and uniform microstructure. This grain refinement is particularly critical for Ti matrix composites. Fine grains not only enhance strength via the Hall–Petch effect but also improve the interfacial compatibility between the reinforcement and the Ti matrix, thereby alleviating stress concentration at the phase boundaries. Yu et al. [56] employed structural design and SPS technology to fabricate titanium alloy Ti64 reinforced with boron (B) and graphene oxide nanosheets (GOs), denoted as (B + GOs)/Ti64 composites. During the sintering process, the SPS temperature was maintained at only 1000 °C (significantly below the β-transus temperature of Ti64, ~1040 °C), resulting in the formation of an optimal amount of TiB (continuous network) and a minimal amount of TiC (interfacial bridging). The continuous TiB network provides long-range load-bearing capacity, while interfacial TiC acts as “nanoscale bridges” to strengthen graphene–Ti bonding—this synergistic phase configuration avoids both excessive brittleness from massive TiC and insufficient strengthening from isolated TiB. The rapid heating and cooling characteristics of SPS effectively suppressed grain growth in fine Ti64 particles while preserving the coarse-grain morphology of larger Ti64 particles. This bimodal grain structure operates synergistically: the fine-grained regions, leveraging the Hall–Petch strengthening effect and the continuous network formed by TiB whiskers, significantly impede dislocation motion and enhance load transfer efficiency [57]; meanwhile, the coarse-grained regions maintain the superior plastic deformation capability of the titanium matrix. The overall microstructure is illustrated in Figure 11. The synergy between SPS and graphene surface modification enables enhanced control over interfacial reactions. The phase transformation temperature of titanium is highly sensitive. While pure Ti undergoes the α → β transformation at ~882 °C, the beta transus for some TMCs can be as high as 995–1000 °C. However, once heated to or above the beta transus, cooling often results in the formation of a lamellar microstructure, which tends to undergo rapid grain growth. This contradicts our goal of achieving fine equiaxed grains. Therefore, in preparing TMCs, it is preferable to select a sub-beta transus temperature as the window for rapid heating and short dwell time. The insufficient atomic diffusion under these conditions can then be compensated for by applying pressure and leveraging prior powder [58].

Although SPS technology has made significant progress in producing high-density materials, its industrial-scale adoption faces challenges, including limited scalability, difficulty in fabricating complex geometries, and issues with process stability and reproducibility. These limitations hinder its widespread use in large-scale manufacturing, especially for aerospace and automotive applications requiring high-precision, large-batch components. Additionally, key sintering parameters—such as heating rate, applied pressure, and holding time—greatly affect the final microstructure and mechanical properties of composites.

**Figure 12:**
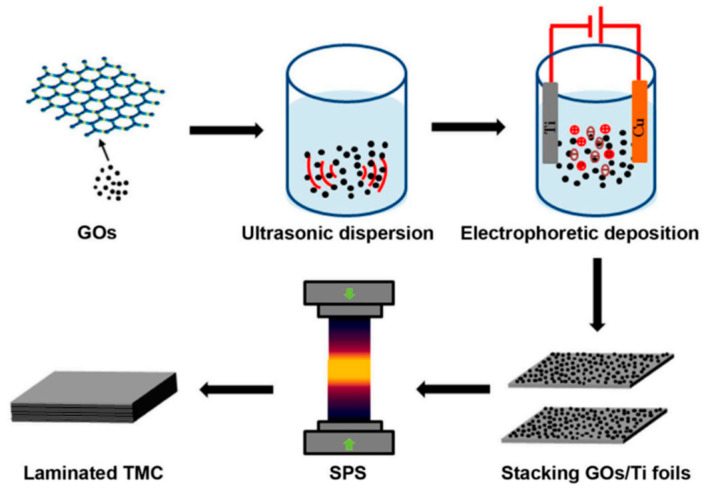
Schematic illustration for the processing route of the in situ TiC/Ti laminated composites [59].

**Figure 13:**
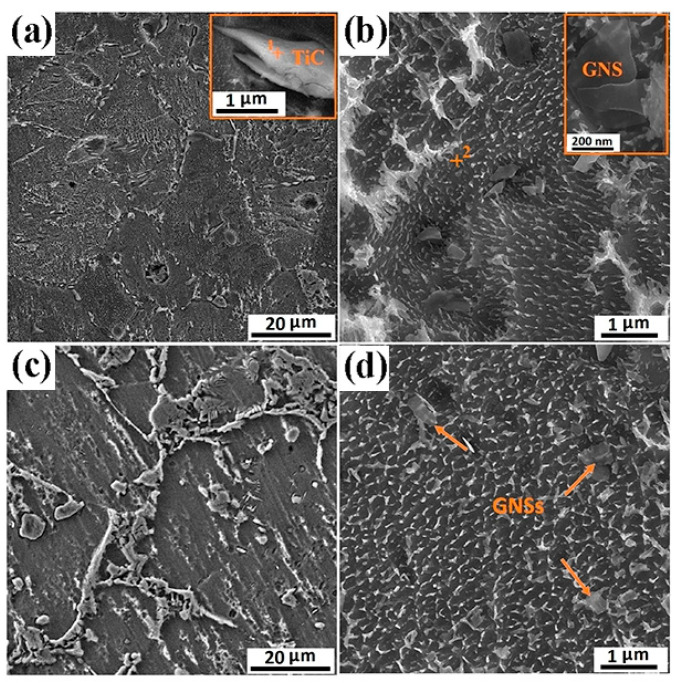
(**a**,**b**) SEM image of Ti/0.5GNSs composite sintered for 1 h, (**c**,**d**) SEM image of Ti/0.5GNSs. Composite sintered for 5 h [25].

Due to the high sintering temperatures characteristic of SPS, titanium readily reacts with graphene at elevated temperatures to form TiC. Excessive TiC formation compromises graphene’s reinforcing effects. Consequently, researchers have devoted extensive efforts to suppress TiC generation during SPS processing. Lei et al. [59] pioneered a hybrid manufacturing approach integrating electrophoretic deposition with SPS. As illustrated in Figure 12, GOs and Al(NO_3_)_3_ were first subjected to ultrasonic dispersion in ethanol. This process utilizes ultrasonic cavitation to exfoliate and homogenize GOs, forming a stable suspension for deposition. Subsequently, the well-dispersed suspension was employed in the electrophoretic deposition process, wherein an applied electric field drives the charged GOs to uniformly deposit onto titanium foil substrates. The deposited 40-layer Ti foils were stacked and subjected to SPS, achieving densification and in situ reactions between Ti and GOs to form TiC. With increasing electrophoretic deposition duration, the deposited amount of GOs on titanium foils rose, resulting in higher volume fractions of TiC reinforcements after SPS. This enhanced material strength at the expense of reduced elongation. Haghighi et al. [25] investigated the effects of GNS content and sintering time on microstructure and mechanical properties. Results revealed that prolonged sintering significantly enhanced material densification and promoted reactions between titanium and GNS to form TiC particles, creating uniformly distributed hard reinforcements. Comparative analysis of Figure 13 demonstrated that increased sintering time elevated the volume fraction of the TiC phase and generated more TiC. Furthermore, compared to the 1 h sintered composite, the microstructure of the 5 h sintered composite contained fewer unreacted GNS. During sintering, the graphite paper used in the SPS graphite mold also causes carbon infiltration into the titanium matrix, creating a surface layer of TiC footprints [60]. This extrinsic carbon contamination is often overlooked but critical—its superposition with intrinsic graphene–Ti reactions exacerbates TiC excess, and the surface TiC layer may cause performance mismatches between the component surface and interior, affecting service reliability. When fabricating GR composites, this extrinsic carbon source superimposes its effects along with inherent graphene reactions, thereby amplifying the risk of an excessive interfacial reaction.

SPS has inherent limitations, such as the inability to fully expel trapped gases during rapid densification, leading to fine, closed pores that can serve as crack initiation sites under stress. While this limits its use, SPS remains a core technology for fabricating high-performance materials.

The development path of SPS technology focuses on overcoming engineering application bottlenecks. Short-term research should optimize large graphite die designs for better temperature uniformity, develop alternative die materials to reduce carbon contamination, and explore integration with thermomechanical processes (e.g., hot rolling, extrusion) to eliminate porosity and reduce internal stresses. Additionally, it is essential to clarify the effects of key parameters on titanium alloys’ microstructure and properties and establish standardized SPS process specifications for specific applications, enabling reliable manufacturing of small-to-medium-batch, high-value components.

### 2.3. Other Processing Techniques

In addition to the extensively investigated PM and SPS techniques, emerging processing routes—such as hot isostatic pressing (HIP), microwave sintering, and selective laser melting (SLM)—have attracted growing research interest owing to their unique processing advantages.

**Figure 14:**
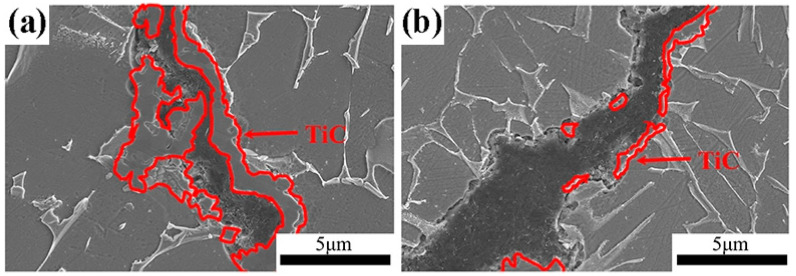
(**a**) HIP 1 wt.% Ni-P@GNF/Ti6Al4V composites, (**b**) SPS 1 wt.% Ni-P@GNF/Ti6Al4V composite [61].

HIP effectively eliminates defects such as pores and microcracks but suffers from prolonged sintering times and poor dispersion of reinforcements. These limitations indicate that extended sintering durations intensify interfacial reactions between reinforcements and the matrix. In fact, the decisive variable is the time–temperature integral rather than pressure per se: once the thermal budget becomes excessive, carbon supply and diffusion promote ripening of interfacial carbides and the networking of GNFs, degrading dispersion and load transfer. Consequently, HIP is primarily suitable for applications with lower GNF content. Cao et al. [61] fabricated GNF/Ti6Al4V composites using both SPS and HIP. As shown in Figure 14, the SPS-processed samples exhibited fine and dispersed TiC particles, while the HIP-processed samples displayed notably coarser and more continuous TiC structures. This microstructural variation is attributed to the HIP procedure, which employed a 900 °C holding time for 3 h. The prolonged sintering during HIP promoted coarsening of TiC and induced aggregation of GNFs. Research by Cai [62] revealed that the SPS process, due to shorter sintering times and rapid cooling rates, resulted in a significantly thinner α-phase compared to HIP-treated specimens. However, the higher sintering temperatures during HIP provided a sufficient driving force for creep deformation and elemental diffusion, resulting in superior high-temperature performance compared to SPS specimens. This trade-off presents a design choice: for creep resistance, HIP’s thicker α phase and stable interfaces are beneficial; for room-temperature strength and toughness, SPS-biased schedules are targeted. Liu et al. [63] employed SPS combined with hot compression deformation to design a two-step HIP process. In the first HIP step, high temperature and pressure facilitated the closure of internal defects such as pores and lack-of-fusion imperfections. The second step, conducted at a higher temperature than the first, promoted grain-boundary migration through elevated heat, leading to the formation of a high proportion of equiaxed grains. This two-step methodology achieved simultaneous improvement in both strength and ductility. HIP offers superior defect elimination and high-temperature performance for graphene-reinforced Ti-based composites but is constrained by interfacial reactions over intensification, while two-step HIP processes break this limitation through staged parameter regulation, forming a complementary technical system with SPS tailored to diverse performance requirements.

The unique ultra-rapid melting–solidification characteristics of Selective Laser Melting (SLM) enable localized in situ synthesis of nanoscale TiC particles while preserving the structural integrity of graphene’s two-dimensional architecture. Yan et al. [13] uniformly dispersed 0.5 wt.% GNSs into Ti-6Al-4V powder via mechanical mixing, followed by the fabrication of high-density GNSs/TMCs through SLM rapid melting–solidification. Compared to conventional thermal processing, the high-energy laser beam instantaneously melted the titanium matrix and promoted rapid cooling, which mitigated excessive reactions between graphene and titanium. This process increased graphene retention by 62% while forming fine TiC particles, simultaneously enhancing interfacial bonding strength by 3.8-fold. The resulting GNSs/Ti64 composites achieved a tensile strength and Young’s modulus values of 1526 MPa and 145 GPa, respectively. However, the ductility of TMCs fabricated by SLM is often compromised due to the excessive size of reinforcements. Joseph et al. [64] addressed this issue by utilizing nano-sized boron nitride (BN) powder as an additive in the SLM process. The high specific surface area of nano-BN facilitated rapid reactions, preventing stress concentration associated with coarse reinforcements. Additionally, BN reacted with molten titanium to form TiB whiskers, whose aspect ratio increased to 400 nm after annealing. The optimized aspect ratio of TiB whiskers enables them to act as load-bearing bridges across matrix grains while allowing plastic deformation of the Ti matrix. This approach successfully resolved the strength-ductility trade-off in conventional TMCs induced by oversized reinforcements.

**Figure 15:**
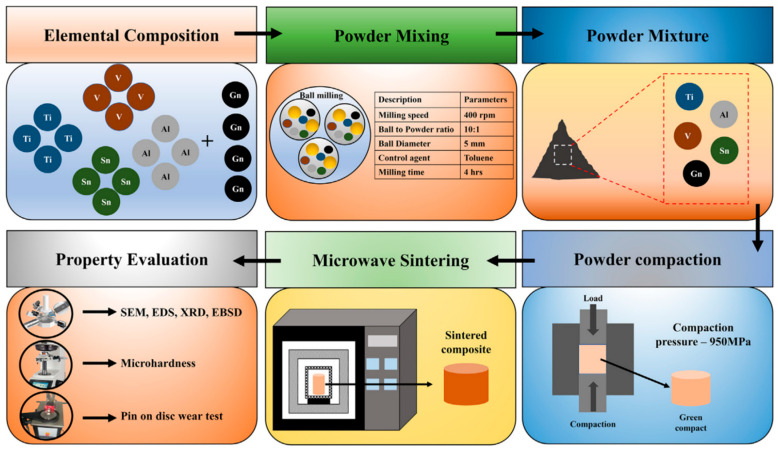
Schematic diagram of microwave sintering method [65].

Microwave sintering significantly reduces sintering temperatures and shortens holding times due to its bulk and selective heating characteristics, thereby suppressing excessive TiC formation from reactions between the titanium matrix and graphene. Akhil et al. [65] incorporated graphene into Ti662 alloy via ball milling and cold pressing, followed by microwave sintering. The detailed process methodology is illustrated in Figure 15. This approach optimized the wear resistance of TMCs. Graphene’s self-lubricating properties and the formation of a mechanically mixed layer effectively suppressed abrasive wear and plastic deformation. The Ti662-Gn composite achieved a microhardness of 514.32 HV, representing a 45% improvement over the matrix alloy, while maintaining high strength and superior tribological properties. Compared to SLM, microwave sintering generated reduced TiC content while delivering enhanced strengthening effects. Table 3 summarizes the key processing parameters and performance results for some of the fabrication methods and reinforcement types mentioned above, comparing graphene-reinforced TMCs under different processes.

As detailed above, each fabrication method offers distinct pathways for incorporating graphene into titanium matrices, with inherent trade-offs between dispersion quality, interfacial reaction control, microstructural refinement, and component geometry complexity. The choice of technique is ultimately dictated by the target performance metrics, cost considerations, and the intended application. To facilitate a clear comparison, the key characteristics, advantages, and limitations of the primary methods discussed are summarized in Table 4.

Current fabrication methods each possess distinct advantages, with some novel approaches achieving uniform graphene dispersion, grain refinement, and even unique laminated or network structures through process optimization. These advancements enhance the synergy between strength and toughness, as well as improve interfacial bonding. However, existing techniques still struggle to fully mitigate graphene agglomeration and interfacial reactions, leading to the formation of brittle TiC phases that compromises reinforcing effects. Consequently, the continuous refinement of preparation processes remains crucial for the development of higher-performance TMCs.

### 2.4. Data-Driven and Machine Learning-Assisted Process Design and Optimization

In addition to mechanistic studies on process–structure–property relationships, modern data-driven approaches such as machine learning are increasingly being applied to optimize composite design and processing. Machine learning models can learn complex relationships between composition, processing parameters, and performance outcomes from experimental data and data in the literature, enabling rapid prediction of mechanical properties and identification of optimal conditions. These data-driven approaches offer significant advantages in accelerating materials development by leveraging large datasets from experiments and simulations.

ML models, such as neural networks, support vector machines, and random forests, have been successfully used to predict key material properties, including strength, toughness, and wear resistance, based on composition, processing conditions, and microstructural features [70,71]. Wu et al. [72] compiled a dataset of 368 data points covering the composition, processing methods, and mechanical properties of various TMCs and demonstrated that ML regression models such as random forest can accurately predict density, hardness, and strength, significantly outperforming empirical estimates and guiding the rapid selection of favorable processing parameters for TMC systems.

Machine learning approaches, particularly Bayesian optimization, have also shown significant promise in multi-objective optimization of composite materials. By iteratively proposing the most informative experimental conditions based on prior results, Bayesian optimization helps optimize multiple processing parameters simultaneously while ensuring that the material properties are enhanced. Nasr et al. [73] used a hybrid artificial intelligence model combining adaptive neuro-fuzzy inference systems and multi-objective particle swarm optimization to optimize the processing parameters for graphene-reinforced Ti-6Al-4V composites. Their approach led to better mechanical properties, including enhanced tensile strength and improved surface quality, by optimizing parameters such as cutting speed and GNP concentration.

Furthermore, recent advancements in deep learning are expected to enhance AI models’ ability to handle more complex, multiscale material systems. Deep neural networks and reinforcement learning are increasingly used to predict and optimize materials properties, offering unprecedented accuracy and efficiency [74]. AI models integrated with traditional physics-based models are expected to offer an even more comprehensive approach for optimizing composite materials. By combining data-driven insights with first-principles modeling, researchers are now able to achieve more accurate predictions of material performance in real-world applications, reducing reliance on experimental testing and accelerating the development of high-performance composites [75].

## 3. Processing Control Strategies for Uniform Dispersion of Graphene

Graphene tends to agglomerate in the metal matrix due to its high specific surface area and strong van der Waals interactions. This aggregation causes stress concentration at the microscale, forming weak interfaces. More importantly, agglomerates hinder dislocation motion and load transfer, reducing graphene’s strengthening efficiency. Once the agglomerate size exceeds a critical threshold, they easily initiate micro-cracks under stress, significantly degrading the composite’s mechanical properties [76,77,78]. Consequently, achieving a uniform and stable dispersion of graphene remains a fundamental challenge in the fabrication of high-performance TMCs.

### 3.1. Powder Homogenization

Ball milling stands as a versatile and scalable mechanical processing technique for fabricating graphene-reinforced TMCs, with its core advantage lying in enabling intimate nanoscale mixing of graphene and titanium powder via intensive mechanical deformation. During milling, repeated collisions between hard balls and powder generate localized high temperature and pressure. This effect activates the surface of the titanium powder and promotes defect formation, while simultaneously introducing high-density dislocations and lattice distortions that provide anchoring sites for graphene [24]. The mechanical force-induced surface activation not only reduces the interfacial energy between graphene and Ti powder but also facilitates physical adsorption or even local chemical bonding, laying a structural foundation for enhanced interfacial load transfer in subsequent sintering.

**Figure 16:**
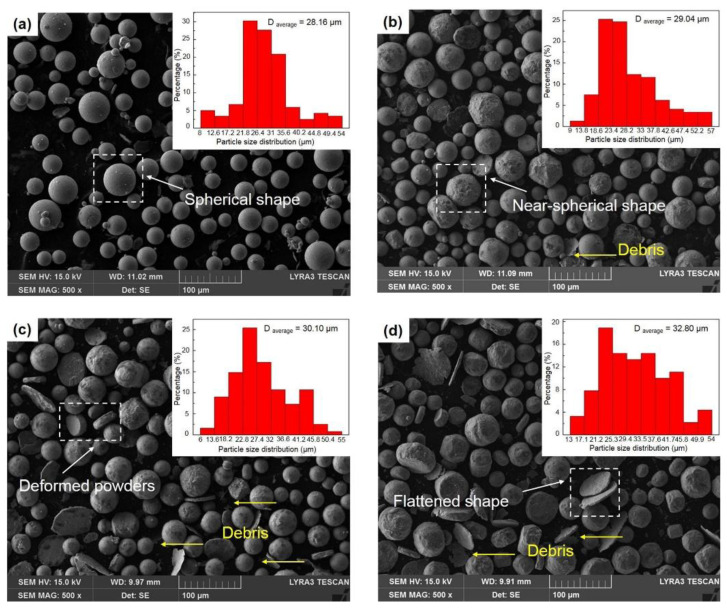
Low-magnification SEM images and particle size distribution of composite powder milled for different times: (**a**) 0 h; (**b**) 5 h; (**c**) 10 h; (**d**) 20 h [34].

**Figure 17:**
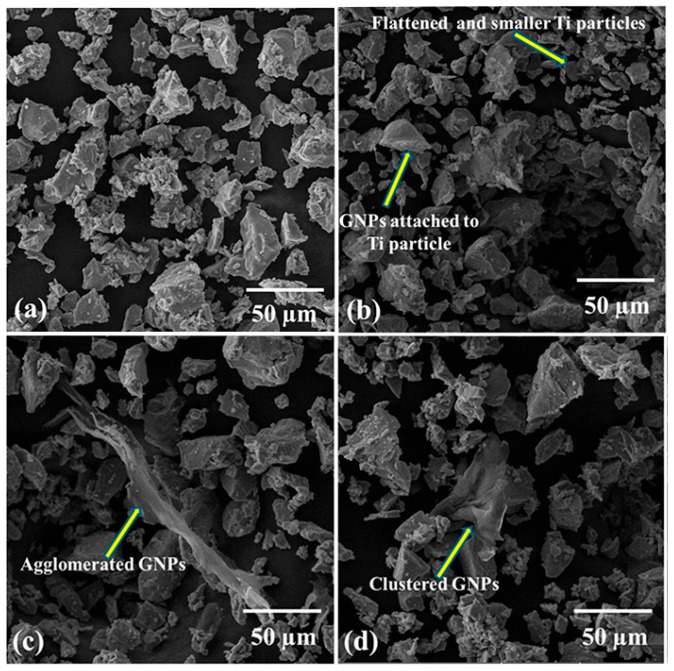
SEM micrographs at magnification of 1 kx showing morphology of (**a**) as-received Ti powder, and 0.25 wt.% GNP-Ti composite powders mixed through (**b**) dry ball milling, (**c**) wet ball milling, and (**d**) rotator mixing [79].

Lin et al. [34] investigated the effects of ball-milling duration on powder properties. Graphene powder and TC4 titanium alloy powder were ball-milled at a ball-to-powder ratio of 2:1 and rotational speed of 300 rpm for durations of 0, 5, 10, and 20 h. As shown in Figure 16, powders milled for 5 h exhibited favorable flowability along with high sphericity, accompanied by uniform attachment of GNPs onto the TC4 particle surfaces. Although extended milling further improved the dispersion of GNPs, it also led to a reduction in powder sphericity and introduced structural defects within the graphene. This highlights a critical optimization trade-off: maximizing GNP dispersion must be balanced against preserving the structural integrity of both graphene and titanium particles, as excessive defects can impair the interfacial load-transfer capability essential for the composite’s mechanical performance. Mahmood et al. [79] achieved material homogenization through three methods: dry ball milling, wet ball milling, and rotary mixing. The corresponding experimental parameters are summarized in Table 5. The design of this table reflects a controlled-variable experimental approach: the upper section lists the distinct mixing parameters for the three methods, aiming to investigate the influence of the initial dispersion mechanism, while the lower section unifies the subsequent compaction and sintering parameters for all samples, ensuring that differences in final performance are primarily attributable to the initial mixing method. Their results revealed method-dependent graphene dispersion mechanisms: dry ball milling employed high-impact forces to flatten titanium particles and promote uniform graphene nanoplatelet (GNP) attachment, achieving the most homogeneous dispersion (Figure 17b); wet ball milling, while partially mitigating agglomeration, exhibited weakened shear and impact forces due to the liquid medium, leading to GNP re-agglomeration likely attributable to fluid drag effects under suboptimal rotational speed or insufficient duration (Figure 17c). In contrast, rotary mixing, conducted without grinding media, demonstrated inadequate dispersion and resulted in heterogeneous graphene distribution (Figure 17d). Many researchers have employed ethanol as a PCA during the ball-milling process. Overall, strengthening efficiency depends not only on dispersion but also on preserving the lateral size and defect state of graphene; over-milling reduces flake aspect ratio and elevates disorder, thereby weakening interfacial load transfer even when surface coverage improves.

**Figure 18:**
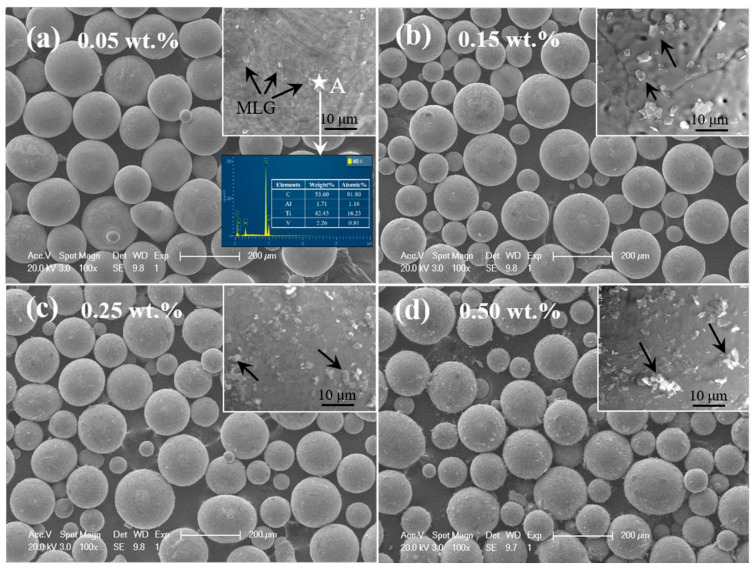
SEM micrographs and inserted higher-magnification images with EDS analysis of MLG/TC4 mixed powders with different MLG contents: 0.05 wt.% (**a**), 0.15 wt.% (**b**), 0.25 wt.% (**c**), 0.50 wt.% (**d**) [43].

As noted earlier, graphene surface modification offers an effective solution to the critical issue of agglomeration induced by strong van der Waals forces. Alternatively, researchers have explored innovative strategies to address this challenge, such as the 3D dynamic mixing method employed by Shang et al. [43]. The mixed powders were rotated at a low speed of 70 rpm within the mixing chamber, producing a three-dimensional tumbling motion that promoted more sufficient contact between MLG and the matrix powders. This method avoided structural damage to MLG and the introduction of impurities caused by traditional high-energy ball milling. Through the synergistic effect of multi-directional collision between powders and surface roughness, when the content of MLG was low (≤0.15 wt.%), uniform coating on the surface of matrix particles was achieved (Figure 18a,b). When the content was too high (≥0.25 wt.%), local agglomeration of MLG occurred (Figure 18c,d), but the overall dispersion was better than that of the traditional method. These results outline a low-shear, low-contamination pathway, whose effectiveness for dispersion, however, diminishes as the MLG content increases. Thus, to achieve uniform coverage without introducing excessive graphene, adopting a carrier-mediated transfer strategy is justified. Based on an indirect mediation mechanism, Mu [80] developed a two-step dispersion process for MLG. In the first step, ultrasonically dispersed MLG suspensions were mixed with agate milling balls serving as an “intermediate carrier”. The choice of agate as the carrier is non-trivial—its moderate surface roughness and chemical inertness enable stable physical adsorption of MLG while preventing carrier-matrix contamination. Through pre-adsorption onto the agate balls, the MLG was transformed from an agglomerated state into a few-layer dispersed state. In the second step, titanium powder slurry was ball-milled with these MLG-coated agate balls, enabling the directional transfer of MLG from the carrier surface to the matrix powder. This two-step approach successfully prevented agglomeration issues that arise from direct contact between MLG and Ti powder. In contrast to Shang’s method, Mu’s approach effectively mitigates the risk of agglomeration through the uniform loading capacity of the milling balls, even at a slightly higher MLG content.

**Figure 19:**
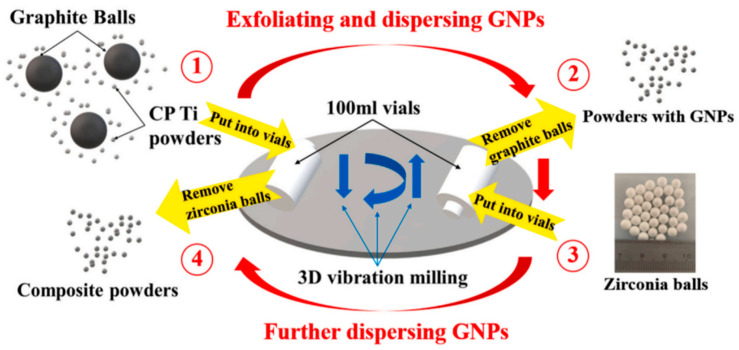
Schematic illustration of the fabrication procedures for in situ GNPs/Ti [81].

Although the ball-milling process enables the mechanical introduction of graphene, the high-energy collisions involved often induce structural defects. In contrast, researchers have established that in situ-exfoliated graphene forms contamination-free interfaces with the titanium matrix, eliminates the necessity for organic solvents, and bypasses the complex procedures associated with pre-dispersion, surface modification, and powder blending required in conventional ex situ approaches. The core superiority of in situ exfoliation lies in its “direct growth” mechanism, whereby GR are generated in the vicinity of Ti particles, minimizing van der Waals-induced agglomeration. Zhang et al. [81] successfully achieved uniform dispersion of GNPs through an in situ exfoliation method from graphite spheres. In this process, commercially pure titanium (CP-Ti) powder and high-purity graphite spheres were subjected to three-dimensional tumbling motions within the milling container. The multi-directional random rotation of graphite balls prevents non-uniform exfoliation caused by unidirectional stress and, in addition, avoids local overheating of the graphite balls. Zirconia balls were subsequently introduced to further enhance the dispersion effectiveness. The entire powder preparation process is schematically illustrated in Figure 19. Notably, the method required no organic dispersing media, and both the Zirconia balls and graphite spheres maintained high purity levels throughout the process.

**Figure 20:**
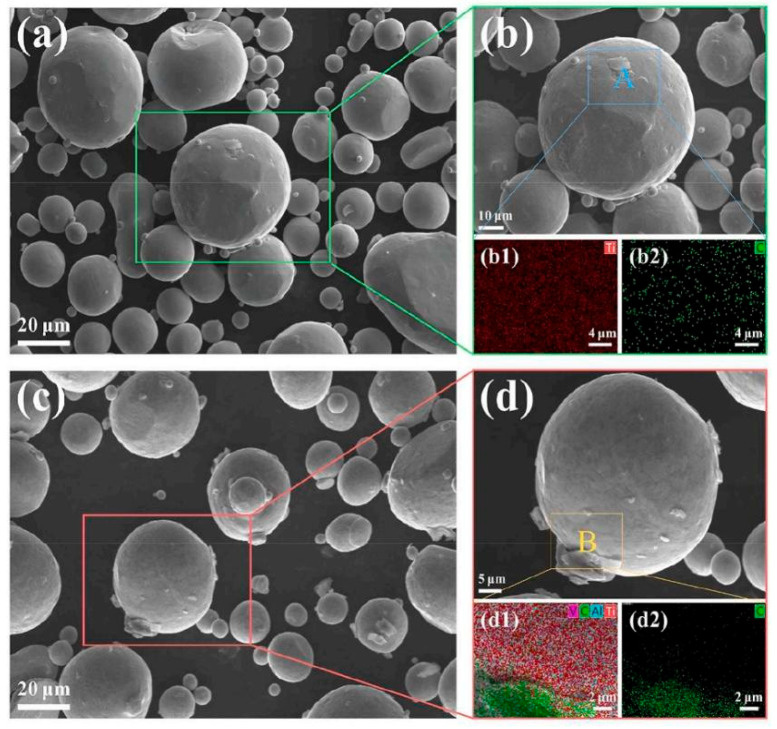
SEM micrographs and high-magnification SEM micrographs of composite powders with (**a**,**b**) 0.13 wt.% and (**c**,**d**) 0.70 wt.% GNPs; element mapping of (**b1**,**b2**) region A and (**d1**,**d2**) B [82].

Building on this in situ exfoliation framework, Zhou et al. [82] optimized the process by integrating 3D vibration ball milling—an approach that improves shear force uniformity compared to tumbling motion—and tailored the dispersion protocol to construct a core–shell structure with practical significance for sintering stability. They prepared mixed powders using a similar in situ exfoliation process: graphene was exfoliated in situ via 3D vibration ball milling at a frequency of 25 Hz, and zirconia balls were incorporated for further dispersion, resulting in the formation of composite powders with a core–shell structure (Figure 20a,c). In contrast to the earlier procedure reported by Zhang et al. [81], the secondary dispersion time in this study was extended by 2 h. The prolonged secondary dispersion facilitates the directional adsorption of exfoliated GNPs onto Ti64 particle surfaces. During the ball-milling process, GNPs grew directly on the surface of Ti64 particles, forming composite powders with Ti64 as the core and GNPs as the shell (Figure 20b,d). This structure effectively prevents the migration and agglomeration of GNPs in subsequent sintering. Such a core–shell topology also constrains lateral graphene mobility during SPS/HIP, stabilizing interfacial continuity. Zhang et al. [83] recently demonstrated that although the two-step 3D vibration-milling method can achieve uniform dispersion of GNPs, the use of zirconia balls during milling introduces additional structural defects. The authors drew on the principle of mild friction between abrasives and the pearl surface in pearl polishing and realized in situ generation of GNPs by frictionally exfoliating the surface layer of graphite balls using a vibration polisher. This method not only attained nanoscale uniform dispersion of GNPs but also significantly improved their structural quality. Specifically, the ID/IG ratio of GNPs@Ti6V4V was approximately 0.39, which is lower than that of GNPs prepared by 3D vibration milling [81,82]. Conversely, the I_2D_/I_G_ ratio reached approximately 0.98, surpassing values reported in previous studies [81,82]. These results suggest that the vibration polishing method facilitates the production of thinner GNPs with fewer defects through mild friction.

Mechanical stirring provides a simple, low-cost, and scalable route for fabricating high-performance TMCs. Through mechanical shear and convective mixing, it effectively breaks up graphene agglomerates and promotes uniform nanoscale dispersion in the titanium matrix. As a low-temperature physical blending process, it avoids the structural degradation of graphene that often occurs during high-temperature treatments. This feature is crucial for preserving graphene’s hexagonal lattice and intrinsic mechanical properties, which are sensitive to thermally induced defects such as vacancies and dangling bonds.

The synergistic combination of mechanical stirring and ultrasonic dispersion is one of the most widely used combinations in current graphene dispersion processes. Liu et al. [39] combined mechanical stirring with hot-press sintering to disperse GO in titanium powder. Commercially pure Ti powder was mixed with a diluted GO solution and subjected to ultrasonic dispersion, followed by mechanical stirring in a water bath to form a semi-dry slurry. The mixture was subsequently dried and ground to obtain a homogeneous composite powder. The ultrasonic treatment leveraged cavitation effects induced by high-frequency vibrations to disrupt van der Waals forces and hydrogen bonding between GO sheets, effectively exfoliating them into single- or few-layer structures within the solution. Subsequently, mechanical stirring, via shear force and convection, prevents re-stratification of titanium powder and GO due to density differences. Moreover, this process enhanced the physical adsorption of GO onto titanium powder surfaces through mechanical action. The synergistic combination of ultrasonication and stirring promoted uniform GO dispersion and strong interfacial bonding with the titanium matrix, respectively; the two steps are complementary rather than interchangeable. While Liu et al.’s work demonstrates the effectiveness of the ultrasound-stirring synergy, the success of such a combined strategy is inherently dependent on the rational matching of process parameters. Minor deviations in key parameters can significantly compromise dispersion quality, as illustrated by Mu et al.’s [84] study. Mu et al. [84] first dispersed GNPs in ethanol via ultrasonication. The resulting suspension was then mixed and stirred with pure titanium slurry for five minutes. However, due to an insufficient ball-milling duration of only 2.5 h, the hybrid powder still exhibited noticeable graphene agglomeration. In contrast, Cao et al. [85] ultrasonically treated GNPs in ethanol for 40 min to form a stable GNP suspension. Then, under mechanical stirring at a rotation speed of 500 rpm, Ti alloy powder was added to the GNP suspension to ensure no agglomeration of GNPs. The comparative results demonstrate that appropriately prolonging ultrasonication time and optimizing stirring conditions can significantly improve the dispersion state of graphene on titanium powder surfaces. Wei et al. [86], during the preparation process, added GO and carbon nanotubes (CNTs) together into an alcohol solution and formed a uniform GO-CNT mixed solution via ultrasonic stirring. The cavitation effect generated by ultrasound further exfoliates CNT agglomerates, while the functional groups of GO are adsorbed on the surface of CNTs, a synergistic dispersion mechanism that not only overcomes the individual agglomeration issues of each reinforcing phase but also constructs a mutually stabilized interface, resulting in a stable and uniformly dispersed hybrid system.

Powder homogenization is essential for fabricating high-performance composites, as it directly determines their final properties and reliability. By achieving uniform mixing at the microscale, this process reduces defects caused by reinforcement agglomeration or segregation. Crucially, it also regulates interfacial reaction kinetics during sintering, preventing both localized carbon enrichment that drives excessive TiC formation and carbon-depleted zones that result in weak bonding. The quality of homogenization governs phase distribution uniformity and subsequent load-transfer efficiency, thereby linking powder processing directly to composite performance. Ultimately, this enables graphene to approach its theoretical strengthening potential and optimizes the overall performance of the composite.

The future development of powder homogenization technology will focus on achieving a balance between dispersion effectiveness, process efficiency, cost control, and scalability. Key research priorities include optimizing the structural and kinematic parameters of low-energy 3D powder mixers to enhance their capability for dispersing higher graphene contents; developing low-cost, low-residue liquid process control agents that are easily removable in subsequent steps; and exploring continuous composite powder preparation techniques such as electrophoretic deposition and spray drying. The core objective is to establish a stable and reproducible composite powder preparation process that can consistently provide high-quality raw materials for subsequent densification.

### 3.2. Powder Densification

Powder densification is a key technological process that converts loose powder into highly densified bulk through a thermal/mechanical/electrical coupling effect [62,87,88]. By optimizing the sintering process and using means such as rapid heating/cooling, low-temperature and short-time sintering, and electric field regulation, the excessive formation of brittle TiC at the interface between titanium and graphene can be effectively inhibited, and graphene agglomeration can be reduced. The advantages of this approach lie in the structural integrity of graphene being preserved by the shortening of the high-temperature exposure time and the uniform dispersion of graphene being promoted and interfacial bonding being enhanced by means of dynamic pressure or an electric field, ultimately improving the mechanical properties of TMCs.

**Figure 21:**
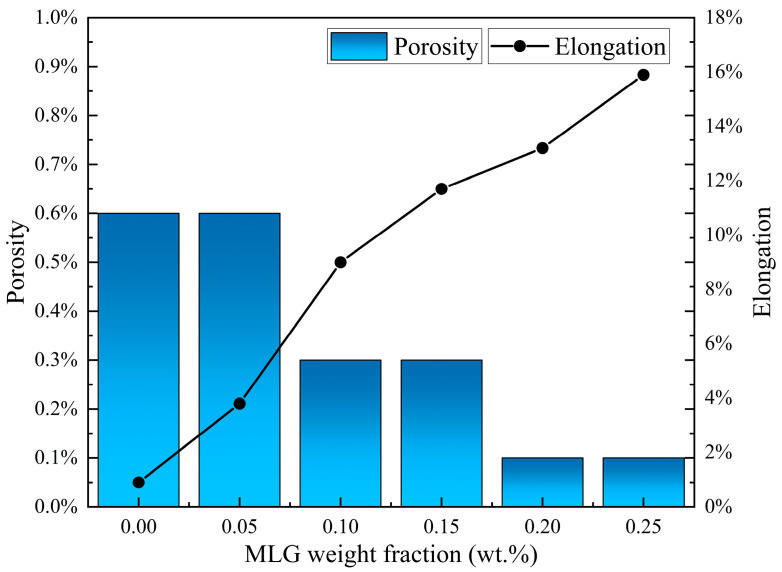
Effect of porosity on elongation in MLG/Ti composites.

Figure 21 shows the effect of MLG content and porosity on the elongation of MLG-reinforced titanium matrix composites. The figure indicates that as the MLG content increases, the porosity slightly decreases (from 0.6% to 0.1%), while the elongation significantly increases (from 0.9% to 15.9%) [43]. This trend suggests that despite the small change in porosity, the material’s elongation exhibits a notable improvement, demonstrating the significant impact of porosity on the material’s ductility. Lower porosity is typically associated with better ductility, which indicates that the reduction in porosity may enhance the material’s plasticity and elongation by improving its microstructure. This further confirms the critical role of uniformly dispersed graphene in enhancing mechanical properties during the densification process.

SPS demonstrates distinct advantages in achieving rapid densification, with the sintering process serving as the critical stage for effectively eliminating pores. In their study on fabricating GO/TC4 composites via SPS, Song et al. [89] elucidated the fundamental mechanisms of SPS in promoting densification. Firstly, the pulsed current generates a high driving force at particle contact points, accelerating the rearrangement of matrix particles. Simultaneously, the applied uniaxial pressure enhances physical contact between reinforcement particles and induces the plastic flow of the matrix particles, thereby accelerating pore closure and densification. Moreover, the characteristic short dwelling time and high heating rate of SPS restrict prolonged high-temperature exposure of the matrix grains and graphene, mitigating adverse long-range diffusion and coarsening, which helps preserve nanoscale reinforcements or control the size of in situ formed phases. This electric-thermal-mechanical multi-field synergy is the core of SPS’s superior densification efficiency compared to conventional sintering. When these three factors are optimally coordinated, SPS achieves high densification under relatively low thermal budgets without causing extensive damage to the graphene structure.

**Figure 22:**
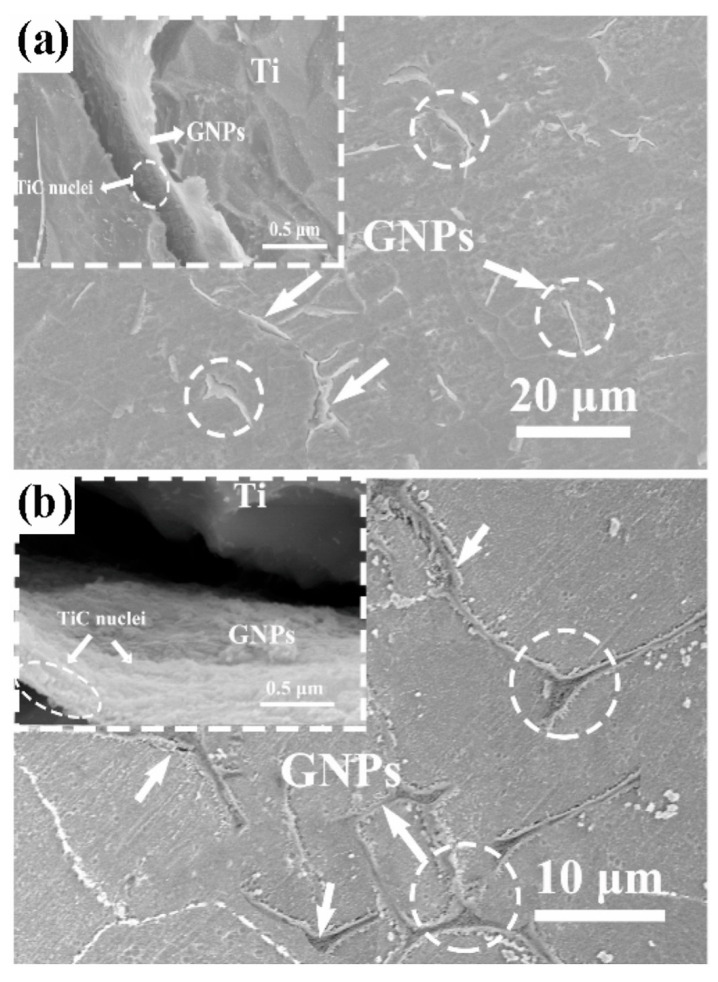
SEM morphologies of (**a**) consolidated GNPs/Ti billet and (**b**) GNPs/Ti-(TiB2) billets by SPS before HT; the insets in (**a**,**b**) are the magnification images of interface structure [90].

Song et al.’s [89] mechanistic insights lay a theoretical foundation for the rational design of SPS parameters, and Liu et al. [90] further verified and expanded the application of SPS in TMCs by optimizing process conditions to balance strength and toughness. Liu et al. [90] employed SPS at 973 K under 300 MPa pressure for a short duration of 5 min. The joule heating and electric field effects generated by pulsed current promoted plastic flow of titanium particles, which effectively suppressed the agglomeration of GNPs. Simultaneously, the high pressure forced GNPs to embed into the titanium matrix (Figure 22), forming a homogeneous network distribution. This mechanical embedding mechanism creates strong mechanical interlocking between GNPs and the Ti matrix, significantly enhancing interfacial load transfer efficiency compared to mere physical adsorption. The short sintering duration also inhibited excessive reactions between GNPs and titanium, reducing premature TiC layer formation. The resulting GNP-(TiBw)/Ti composites achieved an ultimate tensile strength of 1789 MPa while maintaining 22% elongation. Although the short-duration ball milling employed by Mu et al. [84] did not achieve complete dispersion of graphene, subsequent processing via SPS and hot rolling induced significant plastic deformation in the material. The rolling process promoted preferential alignment along the rolling direction, and the flow of metal particles helped fill pre-existing pores, leading to a notable reduction in porosity. Given the susceptibility of both titanium and graphene to oxidation at elevated temperatures—a process that promotes pore formation—Hu et al. [91] introduced an argon shielding atmosphere during laser sintering to isolate the materials from oxygen, effectively suppressing oxidative reactions. This protective measure ultimately enabled the fabrication of a Ti–graphene composite with a highly dense microstructure. However, rapid sintering technology demonstrates high sensitivity to process parameters, complicating precise dynamic regulation of the temperature field. This limitation may induce localized thermal accumulation and provoke secondary agglomeration of graphene. Furthermore, the technique currently encounters scalability constraints and exhibits restricted adaptability for manufacturing geometrically complex components. Addressing these limitations requires the development of intelligent process control systems and innovative mold design.

**Figure 23:**
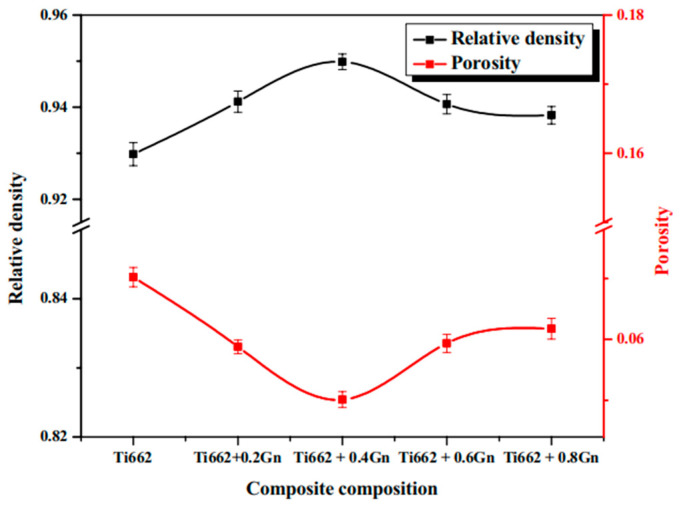
Relative density and porosity of Ti662 + xGn composites Ti662 + xGn [92].

Akhil et al. [92] studied the corrosion behavior of Gn/Ti662 composites and analyzed their densification. As shown in Figure 23, the relative density curve exhibits a distinct peak at approximately 0.4 wt.% Gn, reaching 98.67%, before declining with further Gn addition. Correspondingly, the porosity shows a minimum (~5.02%) at this same composition. This trend is governed by the competing effects of graphene. At low contents (≤0.4 wt.%), uniformly dispersed Gn sheets improve heat distribution and particle rearrangement during microwave sintering, promoting densification and pore sealing. However, higher Gn contents (0.6 wt.% and 0.8 wt.%) led to a reduction in densification. The subsequent decline is attributed to the agglomeration of excess graphene, which creates rigid barriers that hinder particle consolidation and pore closure, while also impairing effective microwave coupling and heating. Consequently, the peak represents an optimal balance between the beneficial role of dispersed graphene and the detrimental effects of agglomeration. These findings highlight the critical role of controlling graphene content in optimizing the densification of the composites. In addition to graphene content and sintering parameters, post-sintering plastic deformation processes (such as high-temperature rolling) also play a pivotal role in optimizing densification by regulating microstructure evolution. Additionally, during high-temperature rolling, the titanium matrix underwent dynamic recrystallization, resulting in the formation of fine equiaxed grains. This recrystallization process, accompanied by grain-boundary migration, contributed to the replacement of prior porous regions with a denser microstructure composed of newly formed grains [84].

The densification process plays a crucial role in significantly improving the relative density of materials. Table 6 compares the characteristics of several primary densification techniques discussed above. Effective densification can eliminate interparticle pores, enabling mechanical properties—such as strength and hardness—to approach their theoretical values. Conversely, insufficient densification leaves residual pores, which act as stress concentrators and sites for crack initiation, ultimately degrading the mechanical performance of the composites. Furthermore, the detrimental effect of residual pores is closely related to their morphology and distribution: irregular, large-sized pores or clustered pores are more prone to crack propagation than small, spherical, and uniformly distributed pores. Thus, controlling pore characteristics serves as an important complement to densification optimization.

### 3.3. Interface Architecture Design

The interface between graphene reinforcements and the titanium matrix plays a decisive role in determining the overall mechanical, thermal, and functional performance of graphene-reinforced TMCs. Owing to the inherent structural and chemical disparity between carbon-based nanomaterials and metallic titanium, achieving a stable and strong interfacial bond remains a fundamental challenge in composite design [93,94,95]. This challenge essentially lies in balancing two contradictory goals: sufficient interfacial reaction to form TiC and minimizing reaction extent to avoid graphene structural degradation—excessive reaction converts sp^2^-bonded graphene into sp^3^-bonded carbides, while insufficient reaction results in weak physical bonding. Therefore, it is essential to achieve a controlled, thin, and uniformly distributed TiC interfacial layer without compromising the structural integrity of graphene, so as to ensure strong bonding.

Researchers have conducted extensive studies to achieve the desired interfacial structure. Decorating graphene with nanoparticles has been demonstrated to mitigate adverse reactions and reinforce bonding. For instance, Yan et al. [96] employed SiC particles to decorate few-layered graphene (FLG), which effectively suppressed the interfacial reaction between FLG and Ti, preserved the sp^2^-bonded structure of FLG, reduced the formation of sp^3^ defects, and simultaneously enhanced interfacial bonding. The SiC decoration acts as a physical barrier that spatially separates FLG from the Ti matrix, regulating the diffusion rate of Ti and C atoms at the interface—this provides a feasible strategy for precisely controlling interfacial reaction intensity. Applying coatings or performing pretreatment on titanium powder surfaces can slow the reaction rate and regulate interfacial phases.

**Figure 24:**
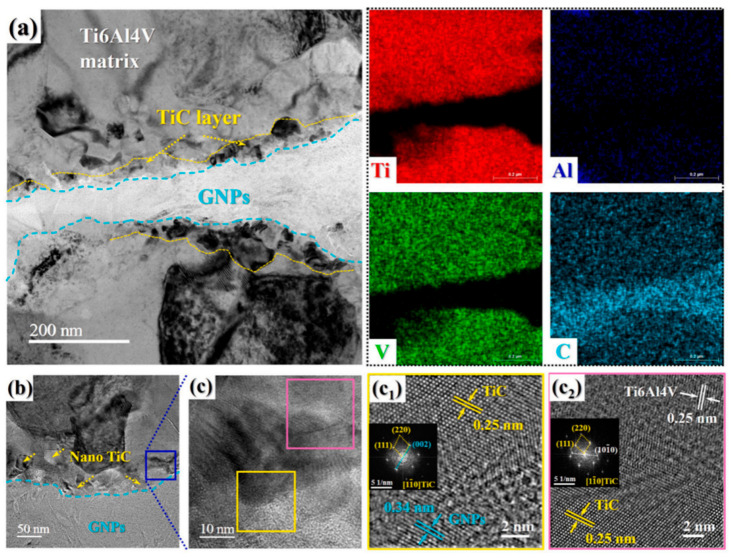
(**a**) TEM BF image at GNPs/Ti6Al4V interface; (**b**) enlarged view of the local area in (**a**); (**c**) high-resolution TEM image of the blue boxed area in (**b**); (**c1**,**c2**) high-resolution TEM images of the GNPs/TiC and TiC/Ti6Al4V interface, respectively, and the corresponding fast Fourier transform image (inset) [97].

Beyond single-component modification, constructing multi-dimensional carbon-based networks is another effective approach to optimize interfacial structure and dispersion stability. Wei et al. [86] combined GO with CNT and applied them in TMCs. The two-dimensional lamellar structure of GO and the one-dimensional tubular structure of CNT are bound through π-π interactions to form a network structure. The GO lamellae insert between CNTs to prevent their re-aggregation, while CNTs are interspersed in the GO lamellae, which enhances the stability of the overall structure. This network structure not only inhibits agglomeration of individual carbon phases but also constructs a continuous load-transfer channel at the interface, enabling efficient stress transmission between the Ti matrix and carbon reinforcements. Feng [97] fabricated a layered composite by alternately stacking pure Ti6Al4V and GNPs/Ti6Al4V layers. During the hot rolling process, by regulating the reaction degree between GNPs and the titanium matrix, a nanoscale TiC layer with a thickness of 20–90 nm was observed on the surface of GNPs via TEM, as depicted in Figure 24. This TiC layer wrapped around GNPs, preventing their sliding or agglomeration. Moreover, the hot rolling temperature of 1173 K exceeded the recrystallization temperature of titanium (approximately 800 K), which induced dynamic recrystallization within the matrix. This process refined the grain structure, increased grain-boundary density, hindered the migration of GNPs, and further stabilized their uniformly dispersed state. Feng’s study highlights the synergy between processing-induced microstructure evolution and interfacial reaction regulation—grain refinement not only improves matrix strength but also provides more grain boundaries to “anchor” GNPs, complementing the bonding effect of the TiC layer. Building upon the concept of TiC-based interfacial bonding, Liu et al. [98] designed a structure similar to that reported by Feng [97]. They further refined this interface by introducing a symmetric sandwich architecture, where GNPs are tightly encapsulated by TiC layers on both sides, forming a TiC/GNPs/TiC configuration. This design not only enhances the interfacial bond strength but also, through the spatial confinement effect of the TiC layers, immobilizes GNPs to prevent their agglomeration. During high-temperature processing, this spatial confinement is particularly crucial for maintaining dispersion—the TiC layers act as “nano-cages” that restrict GNP mobility, thereby preventing their re-agglomeration driven by surface energy minimization. Furthermore, constructing three-dimensional graphene networks physically inhibits graphene migration and agglomeration within the titanium matrix through mechanical interlocking. This strategy shifts the interfacial design focus from point-to-point bonding to network-scale interlocking. Consequently, the interface evolves from mere graphene–Ti contact into a deliberately engineered zone comprising coatings, reaction products, nanostructured interlayers, and networked reinforcements.

**Figure 25:**
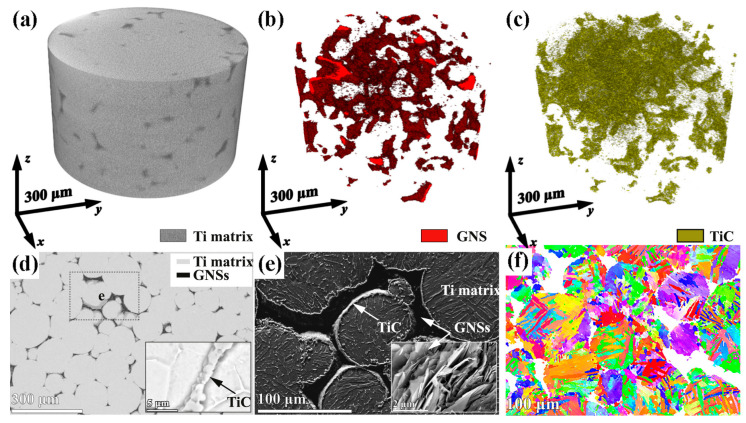
Microstructure of GNS/Ti64 composites. (**a**) The three-dimensional density contrast image with (**b**) corresponding 3D-distributed image of GNSs and (**c**) TiC. (**d**) BSE and the SEM image (**e**) with the corresponding EDS mappings (**f**) [45].

Conventional interfaces frequently exhibit either inadequate bonding strength or excessive reaction phases that degrade the structural integrity of graphene. To resolve this dichotomy, novel interface designs strategically combine physical confinement with regulated chemical bonding, establishing structurally robust and mechanically optimized interfaces. Current research prioritizes the development of multiscale hybrid and sandwich-like interfacial architectures to simultaneously stabilize graphene within the titanium matrix and enhance interfacial load-transfer efficiency. Yan et al. [45] designed a 3D network structure where GNS were distributed at the primary particle boundaries of titanium powder, forming a quasi-continuous network, as illustrated in Figure 25a–c. The wrinkled and stacked morphology of the GNS established multiscale geometric interlocking with the titanium matrix. Because planar graphene is prone to sliding under shear stress, the wrinkled structure can resist shear deformation, which is crucial for improving interfacial toughness. This multiscale geometric interlocking transforms the interfacial interaction mode from point contact to surface interlocking, which not only strengthens the resistance to interfacial separation but also expands the effective load-transfer area between GNS and the matrix. This network structure created an anchoring effect at the microscopic scale, restricting the relative sliding between the matrix and GNS and inhibiting the formation of isolated agglomerates. Submicron TiC particles were observed in the regions surrounding the GNS, as shown in Figure 25d,e. High-Resolution Transmission Electron Microscopy Images depicted in Figure 25f further confirmed that these interfacial nanocrystals were TiC, indicating a dense and tough interfacial bond between the GNS and the titanium matrix. The coexistence of 3D network geometric interlocking and TiC chemical bonding forms a dual-secure interface—geometric interlocking provides immediate mechanical constraint, while TiC realizes chemical bonding for efficient stress transmission. This multiscale TiC design is scientifically rigorous: nano-TiC targets the intrinsic interplanar agglomeration tendency of GR by filling gaps, while submicron-TiC addresses the macroscopic sliding issue through mechanical anchoring—their synergistic steric hindrance effect achieves full-dimensional regulation of graphene behavior from micro to macro. This steric hindrance effect not only inhibits the agglomeration tendency of graphene but also achieves uniform dispersion through the topological constraints of the 3D network architecture.

Simulation technologies demonstrate significant value in interface design, particularly for dispersion control strategies, by accurately predicting interactions and interfacial behavior between graphene and titanium matrices. Simulation technologies compensate for the inherent limitations of experimental methods in capturing atomic-scale dynamic processes—unlike experiments constrained by spatial–temporal resolution, molecular dynamics simulations can visually track the diffusion, adsorption, and bonding evolution of graphene at the titanium interface, providing quantitative data that lays the foundation for rational interface design. Molecular dynamics simulations can trace graphene diffusion pathways within titanium substrates, identifying agglomeration-prone zones (e.g., grain boundaries, dislocation pile-up sites) to enable targeted interface optimization. However, conventional studies lack molecular dynamics investigations into the interfacial mechanical properties of graphene–titanium composites, which limits the development of accurate composite interface models.

**Figure 26:**
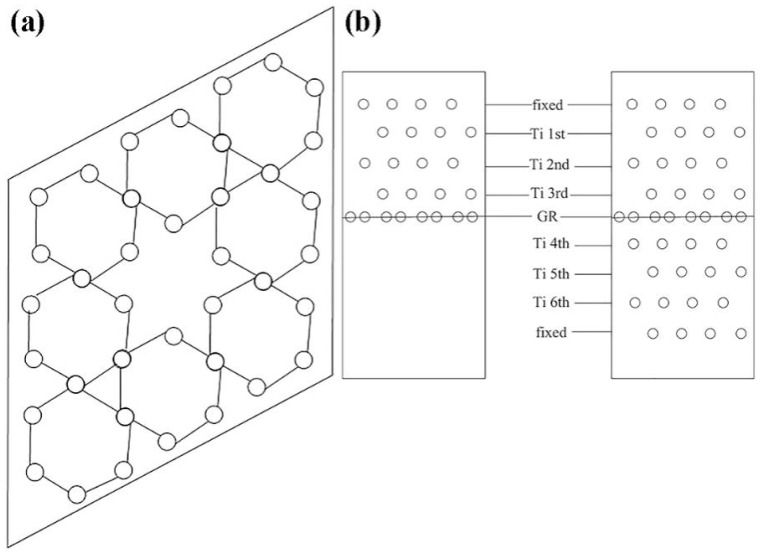
(**a**) Graphene/titanium single interface model and (**b**) graphene/titanium dual interface model [99].

**Figure 27:**
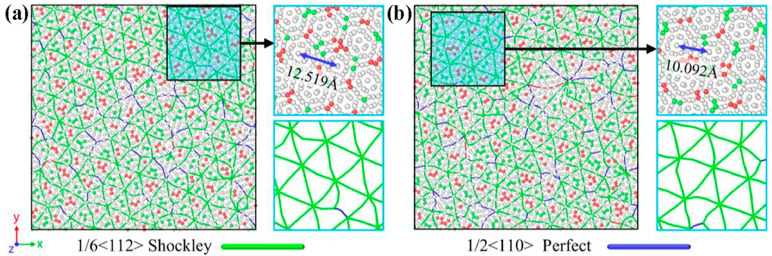
(**a**,**b**) are cross-sectional diagrams of the dislocation network, among which are the Shockley dislocation (green) and the Perfect dislocation (blue) [100].

Zhang et al. [99] proposed a method using the beam search algorithm, which established single-interface (Figure 26a) and double-interface models (Figure 26b). In the double-interface model, graphene is sandwiched between two layers of titanium matrix, forming a ‘sandwich’ structure. The crossed-circle symbols in the interface diagrams indicate regions of enhanced electron overlap and covalent interaction between graphene and adjacent titanium layers. This configuration restricts the migration of graphene due to the steric hindrance effect of the titanium layers. Moreover, the double interface is more stable, with stronger covalent interactions between graphene and the adjacent titanium atomic layers. The double-interface design’s ingenuity lies in its synergistic integration of physical confinement and chemical bonding—steric hindrance from titanium layers directly suppresses graphene migration during processing, while enhanced covalent bonding optimizes load-transfer efficiency under service conditions. While Zhang et al.’s [99] study focuses on static interface structure and stability, Gao [100] extended simulation research to dynamic microstructural evolution during composite processing, revealing the intrinsic correlation between interface behavior and bulk mechanical properties. Gao [100] established an alternately stacked Gr/TiAl laminar composite model. Molecular dynamics simulations revealed that the composite is more prone to crystallization after rapid solidification. As shown in Figure 27, a dislocation network dominated by Shockley dislocations (with Perfect dislocations being secondary) forms, accompanied by a periodic hexagonal Moiré superlattice. The Moiré structure is essentially a periodic dislocation network caused by the lattice mismatch between graphene and TiAl. It increases the contact area and bonding strength between graphene and the matrix, significantly enhances interfacial shear resistance, and hinders the sliding of graphene along the direction parallel to the interface.

All the above simulations assume that the graphene layers are perfect and defect-free (e.g., without wrinkles or oxidation). This idealized assumption deviates from practical preparation scenarios, as defects such as wrinkles (induced by processing stress), oxygen-containing functional groups, and carbon vacancies (from high-temperature interfacial reactions) are almost inevitable—different types and densities of defects can drastically alter interfacial energy, bond configuration, and load-transfer paths, leading to deviations between simulation predictions and experimental results. However, in actual preparation, defects may be introduced during preparation due to interfacial reactions or processing damage, which affects interfacial bonding strength and load transfer efficiency.

Molecular dynamics simulations by Fonseca et al. [101] showed that when double-interstitial defects (formed by two extra carbon atoms) are present on graphene surfaces with substrate curvature, stress-induced out-of-plane displacement occurs at the defective carbon atoms. The curved substrates deform the graphene, weakening the interatomic C-C bonds, while the defects themselves lead to local structural loosening. These dual effects collectively reduce the energy barrier for carbon atom migration. This reduction in migration energy barrier is not merely a structural phenomenon but a key kinetic driver for interfacial chemistry—moderate barrier lowering can promote the formation of uniform, thin TiC layers that enhance bonding, while excessive reduction may trigger uncontrolled carbon diffusion, leading to thick, brittle TiC aggregates that degrade composite toughness. Additionally, the formation of TiC is influenced by the crystal structure.

**Figure 28:**
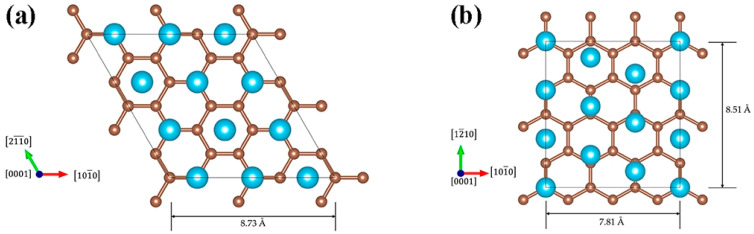
Schematic diagram of the relationship between Ti/graphene interface sites with different crystal structures; (**a**) α-Ti/graphene; (**b**) β-Ti/graphene [10].

First-principles studies by Han et al. [10] demonstrated that the body-centered cubic (BCC) structure of β-Ti has a higher atomic packing density and symmetry (Figure 28a), forming semi-coherent interfaces with graphene. Conversely, the hexagonal close-packed (HCP) structure of α-Ti exhibits better lattice matching with graphene (Figure 28b). However, the strong chemical reactivity of its close-packed (0001) planes promotes significant Ti-C bond formation. This crystal structure-dependent interfacial behavior provides clear design guidance: β-Ti is preferred for scenarios requiring suppressed interfacial reactions to preserve graphene integrity, while α-Ti can be leveraged when stronger Ti-C bonding is prioritized—with the caveat that reaction extent must be constrained to avoid excessive TiC formation. The authors also noted that vacancy defects in graphene increase unsaturated chemical bonds in neighboring carbon atoms. These dangling bonds provide binding sites for titanium atoms and accelerate Ti-C bond formation. Consequently, precise control over graphene defect concentration, substrate crystallography, and surface morphology becomes essential for tailoring interfacial chemistry. Crucially, these factors are not independent—substrate curvature can induce defect formation, and crystal structure affects the reactivity of defect sites—thus synergistic regulation is necessary to achieve optimal interface performance. Understanding these atomic-level interactions provides critical insight into the design of stable, semi-coherent Ti–graphene interfaces, offering a theoretical foundation for achieving optimized interface bonding and enhanced mechanical reliability in graphene-reinforced TMCs. The atomically synergistic modulation of graphene defects, the crystal structure, and the surface morphology of the titanium matrix is central to the precise tailoring of Ti–graphene interfacial chemistry. By balancing carbon migration kinetics with Ti-C bonding strength, it provides the key theoretical foundation for unifying high bonding strength and structural stability in composites.

Future interface structure design will increasingly focus on achieving stable and reliable interface control within existing process windows. On one hand, precise regulation of ball-milling processes can create tunable nanoscale roughness or reactive sites on titanium powder surfaces, thereby enhancing mechanical interlocking effects. On the other hand, in situ reactions during sintering can be utilized to generate TiB whiskers or nanoscale TiC particles, which can be directed to enrich the interface region to provide effective physical pinning. Additionally, the feasibility of introducing thin, dense oxide or nitride barrier layers on graphene surfaces should be evaluated to delay titanium–carbon reactions. These strategies must maintain good compatibility with existing powder metallurgy processes, aiming to achieve controllable construction of interface structures and stable performance reproduction.

## 4. Inhibition and Engineering of Graphene–Titanium Interface Reactions

The interfacial interaction between graphene and the titanium matrix plays a pivotal role in determining the mechanical properties and structural stability of graphene-reinforced TMCs. This interaction is fundamentally governed by a delicate balance: sufficient chemical bonding is necessary to ensure efficient load transfer from the matrix to the reinforcements, yet it must be carefully controlled. Excessive interfacial reactions, such as the uncontrolled formation of thick, brittle TiC layers or the chemical degradation of graphene, will severely compromise their intrinsic reinforcing effects. A thick, continuous TiC layer not only consumes the structurally sound graphene but also creates a brittle interface prone to crack initiation under stress. Conversely, a weakly bonded interface fails to transfer load effectively, rendering graphene reinforcement inefficient. Therefore, achieving an optimal interface—often characterized by a controlled amount of in situ formed nano-TiC alongside structurally preserved graphene—is paramount to harnessing the full potential of these advanced composites.

### 4.1. Mechanisms and Challenges of Interface Reactions in Graphene/Titanium Composites

The interfacial reaction between graphene and the titanium matrix is defined by the in situ formation of TiC.

During high-temperature processing (e.g., sintering, hot extrusion, or additive consolidation), carbon species released from graphene edges/defects or partial decomposition diffuse into titanium and react to form TiC (Ti + C→ TiC). This process is driven by the interdiffusion of carbon and titanium atoms under thermodynamic and kinetic conditions favorable for TiC nucleation and growth.

**Figure 29:**
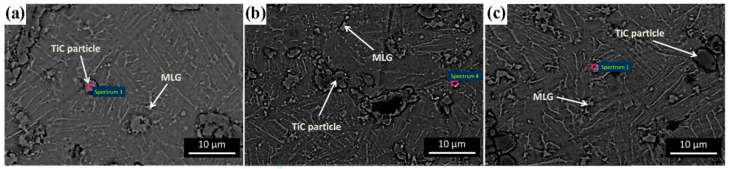
Scanning electron microscope images of (**a**) Ti64–0.4 wt.% MLG (**b**) Ti64–0.8 wt.% MLG (**c**) Ti64–1.2 wt.% MLG [21].

**Figure 30:**
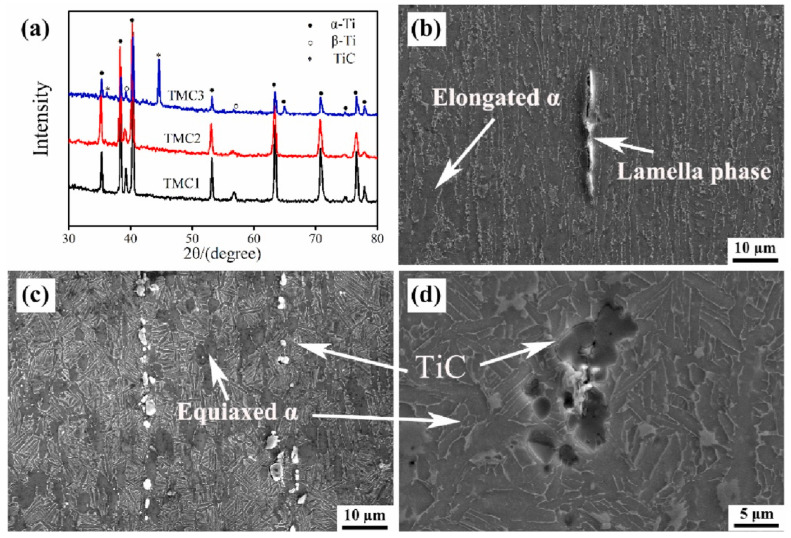
XRD patterns of TMCs (**a**); SEM of TMC1 (**b**), TMC2 (**c**), TMC3 (**d**) [102].

Sharma et al. [21] demonstrated the in situ reaction between the Ti matrix and C under high temperature during the SPS process in an experiment preparing MLG/Ti64 composites. The author found that C atoms at the edges and defects of MLG preferentially dissociated and diffused to the Ti grain boundaries. Subsequently, the C atoms bonded with Ti at the interface to form TiC nuclei. Moreover, as shown in Figure 29a–c, the concentration of TiC in the matrix is dependent on the amount of MLG reinforcements in the mixture. High temperatures provide the required thermal activation for carbon and titanium atoms to overcome diffusion barriers, thus promoting the in situ formation of TiC at the interface. However, excessive temperatures may also lead to undesirable coarsening of TiC particles or degradation of graphene’s structural integrity. Hou et al. [102] further elucidated the quantitative relationship between processing temperature and the formation/growth of TiC. They subjected pre-sintered green compacts to hot extrusion at 900 °C (TMC1), 1000 °C (TMC2), and 1100 °C (TMC3) respectively. The study found that the sample extruded at 900 °C (TMC1) was mainly composed of elongated α grains and lamellar β phases, with no obvious formation of TiC particles (Figure 30a,b). In contrast, the samples extruded at 1000 °C and 1100 °C exhibited a substantial increase in the average size of the TiC particles, with prominent TiC peaks observed, which is shown in Figure 30c,d. The driving force for this process comes from the high diffusion ability of atoms at high temperatures, enabling Ti and C to overcome the reaction energy barrier and form stable TiC. Kvashina et al. [103] confirmed that the critical temperature threshold for the Ti-C reaction lies within this range through pressureless sintering of titanium and graphite at 900–1000 °C. Graphene defects markedly amplify interfacial reactivity. At elevated temperatures, partial graphene decomposition can release more active carbon, while edge sites and vacancy-type defects provide energetically favorable nucleation sites and diffusion pathways for Ti–C bonding [104,105]. Building upon this understanding, Han et al. [10] further observed that vacancy defects expose dangling C bonds, which intensify Ti-C covalent interactions, playing a crucial role in the formation of TiC. Excessive defects disrupt graphene’s structural integrity, converting discrete nanoscale TiC into continuous brittle layers—highlighting the need for defect density optimization to balance reactivity and structural preservation.

**Figure 31:**
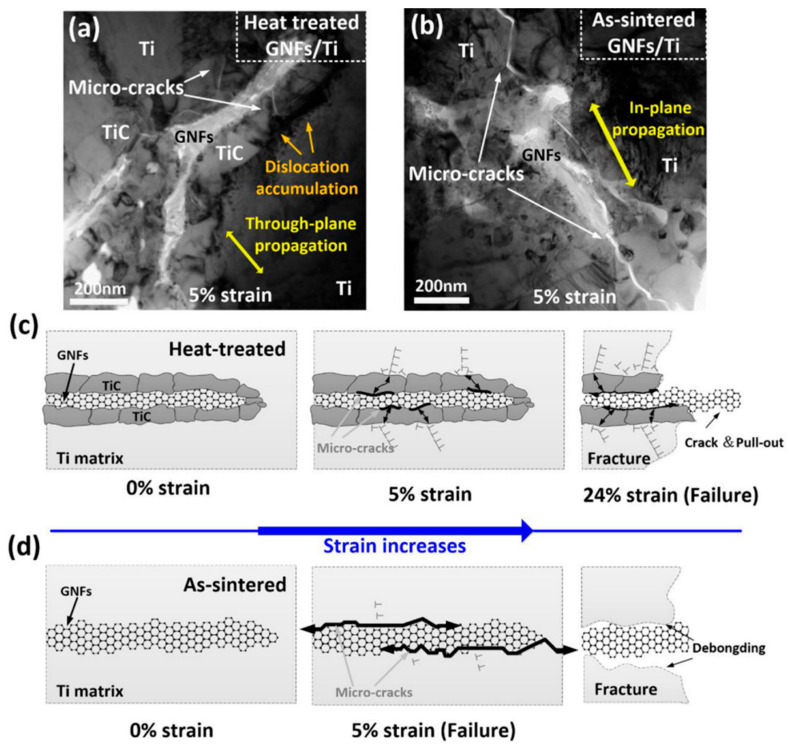
(**a**,**b**) TEM analyses of interfacial area in heat-treated and as-sintered GNF/Ti composites at 5% tensile strain; (**c**,**d**) schematic illustration of the interfacial failure process in heat-treated and as-sintered GNF/Ti at various tensile strains [106].

The design objective is not to completely eliminate TiC but instead to confine its morphology within an ideal window: finely distributed and discontinuous TiC or an ultrathin, non-continuous layer can enhance bonding and load transfer, while a thick, continuous TiC interfacial layer (or a TiC network connected along grain boundaries) typically leads to embrittlement and premature interfacial cracking. An optimal TiC layer thickness can enhance interfacial chemical bonding and improve load transfer efficiency, while avoiding the embrittlement and stress concentration issues caused by an excessively thick layer. Mu et al. [106] subjected the mixed powder of GNFs and titanium to SPS sintering, followed by heat treatment to induce a reaction between part of the GNFs and titanium, forming a chemically bonded TiC interface layer approximately 87 nm. Tensile tests on both as-sintered and heat-treated TMCs revealed that in the heat-treated TMC, cracks were pinned by the multi-sized grains in the TiC layer. When microcracks propagated into the TiC layer, they were forced to propagate along the TiC grain boundaries (Figure 31a), allowing the material to withstand strain up to 24% before failure. This multiscale grain architecture creates a hierarchical energy dissipation mechanism, where fine grains provide strength while grain boundaries enable toughening through crack deflection. In contrast, the TiC layer in the as-sintered TMC was only 2–5 nm thick, and the weak interfacial bonding could not prevent crack propagation, leading to rapid crack propagation along the GNF/Ti interface, shown in Figure 31b. Moreover, the heat-treated TMC withstood strain until failure at 24% strain (Figure 31c), whereas the as-sintered TMC without heat treatment, lacking the influence of the TiC interface, failed only after 5% strain (Figure 31d). This phenomenon can be scientifically explained by the crack pinning and deflection mechanism in fracture mechanics: the multi-sized TiC grains increase the resistance to crack propagation—fine grains hinder crack movement through the Hall–Petch effect, while the grain-boundary interface forces cracks to deflect, consuming additional fracture energy.

While a controlled amount of TiC can enhance interfacial bonding, excessive TiC formation typically produces a thick, brittle interlayer that compromises ductility and fracture toughness [42,107].

During the hot rolling of FLG-reinforced Ti64 composites, Mu et al. [108] applied short-term heat treatment (450 s) to sintered TMCs, forming a continuous nanoscale TiC layer (~74 nm) that achieved strong interfacial bonding between GNFs and the titanium matrix. Extending treatment to 600 s thickened the TiC interface to 236 nm. However, this thickness increase failed to deliver the expected reinforcement; instead, excessive TiC adversely affected mechanical properties and the continuous thick TiC layer became a rapid crack propagation channel rather than a crack barrier. Markovsky et al. [109] demonstrated that an increase in TiC content leads to a significant reduction in both tensile strength and ductility. At 5 vol.% TiC, the yield and tensile strengths plateaued at 708 MPa, while the ductility nearly vanished (≤0.1%). With 20 vol.% TiC, the UTS dropped to 567 MPa, accompanied by a transition to brittle fracture behavior. Although TiC particles provided better reinforcement under compression loading, their inherent brittleness limited extreme dynamic applications. Chang et al. [110] fabricated TiC/graphene/Ti6Al4V composites via laser powder bed fusion at 400 °C, 500 °C, and 600 °C. The incorporation of graphene resulted in microstructural refinement of acicular α′-martensite in the Ti6Al4V matrix, and the uniform dispersion of TiC particles contributed to a reduction in porosity. As a result, the composites produced at all three temperatures exhibited enhanced tensile strength and microhardness compared to the unreinforced Ti6Al4V alloy. However, increasing temperatures accelerated dislocation motion, promoting nucleation/coalescence of micropores on matrix surfaces. At 600 °C, the minimal tensile strength increment reached only 538 MPa—merely 76 MPa above the matrix.

In summary, interface reactions in graphene-reinforced TMCs are thermodynamically favorable and inevitably lead to TiC formation; the key challenge is to engineer TiC morphology and continuity to achieve simultaneous strengthening and toughening. This balance can be understood through three coupled control knobs: (i) carbon activity (defect density and chemical state of graphene), (ii) diffusion budget (time–temperature history and heating/cooling rate), and (iii) contact geometry (dispersion uniformity and local carbon enrichment). Future studies should therefore quantify TiC thickness, continuity, and size distribution together with graphene integrity and correlate these descriptors with fracture-path statistics and property scatter, enabling predictive design of interfacial architectures.

### 4.2. Interface Reaction Inhibition Strategies in Graphene-Reinforced Titanium Matrix Composites

Controlling or suppressing interfacial reactions continues to represent a persistent scientific challenge. From a thermodynamic perspective, the driving force for TiC formation is so large that it is extremely difficult to completely prevent the reaction between graphene and the titanium matrix. Although fully predictive and universally applicable control over graphene–Ti reactions is still lacking, researchers have developed integrated strategies—combining interlayer design, alloying additions, and process-window optimization—to tune interfacial reactions and balance bonding strength with graphene integrity.

**Figure 32:**
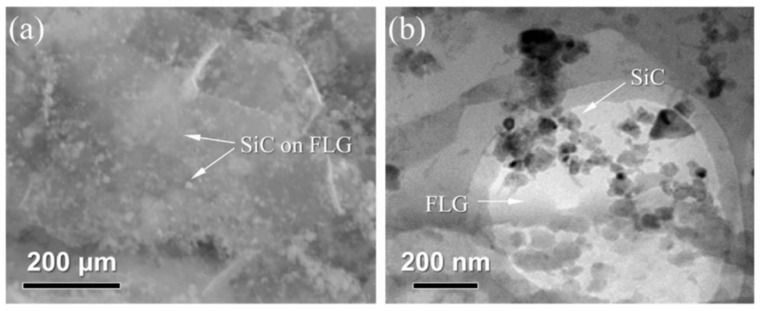
Morphology of FLG decorated with SiC nanoparticles in SEM image (**a**) and bright TEM image (**b**) [111].

Interfacial regulation is commonly achieved via two complementary routes: chemical functionalization/defect-state tuning (altering carbon activity and diffusion) and interlayer/coating engineering (reducing direct contact and introducing diffusion barriers). In practice, the latter—including ceramic nanoparticle decoration and metallic electroless coatings—has proven particularly effective for moderating Ti–C reactions while maintaining load transfer. In their study, Yan et al. [111] uniformly deposited SiC nanoparticles onto the surface of FLG through solution dispersion, as illustrated in Figure 32. The SiC nanoparticles primarily act as spatial spacers and local diffusion barriers, reducing direct FLG–Ti contact; this interlayer can modify the local interfacial chemistry and kinetically retard long-range carbon diffusion from FLG into the Ti matrix. The preferential formation of discrete TiC nanoparticles from the reaction between SiC and Ti kinetically impedes carbon diffusion from FLG into the Ti matrix. Furthermore, the released silicon atoms form a Ti-Si solid solution, which suppresses carbon migration through the solvent effect and enhances interfacial hardness simultaneously. Ge et al. [41] employed electroless nickel plating to coat GNFs. During subsequent heat treatment, the Ni layer serves as a protective barrier, mitigating reactions between GNFs and titanium and preserving the GNFs’ nanostructure. Significantly, Ni diffuses into the Ti matrix, forming feathery NiTi_2_ intermetallic with specific crystallographic orientation, which markedly enhances the load-transfer capacity across the interface. This dual-function design (barrier + strengthening) provides a promising approach for interface regulation, as the NiTi_2_ intermetallic can absorb energy during deformation, effectively preventing crack propagation along the interface. Zhang et al. [26] utilized an electroless Ni–P coating on graphene to modify the graphene–titanium interface. The deposited Ni-P layer reduces direct contact between graphene and titanium, and its high chemical inertness lowers interfacial reactivity, thereby suppressing excessive TiC formation. Concurrently, the catalytic effect of nickel promotes the formation of nano-sized TiC particles, which enhances interfacial strength without sacrificing toughness. The approach by Ge et al. [41] allows for moderate interfacial reactions to form a beneficial interlayer, whereas Zhang’s [26] method prioritizes stabilizing the interfacial structure and preserving the intrinsic properties of graphene. Collectively, these two strategies demonstrate that by designing rational metallic interlayers, a balance can be achieved between interfacial reactivity and structural stability. Processing parameters (temperature, time, pressure, heating rate, and atmosphere) ultimately determine whether the designed interlayer/alloying strategy succeeds, because they govern the diffusion budget and the real interfacial contact state. Temperature and dwell time control Ti–C reaction kinetics and TiC coarsening, whereas pressure promotes pore closure and plastic flow but also increases the effective contact area and diffusion accessibility. Therefore, interlayer design must be interpreted together with the processing window, rather than as a standalone solution.

**Figure 33:**
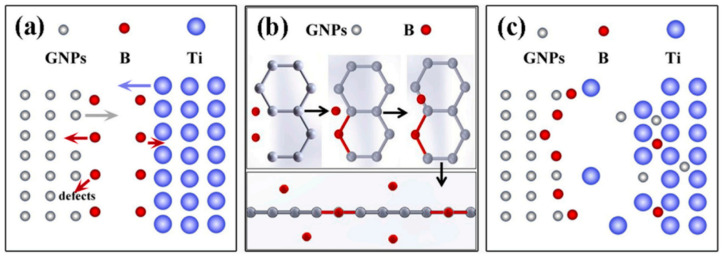
The initial diffusion schematic of C, B and Ti atoms (**a**); the schematic of B adsorption on GNPs (**b**); the final diffusion schematic of C, B and Ti atoms (**c**) [31].

B is a highly reactive boride-forming element in Ti-based systems and can significantly modify interfacial chemistry and reaction pathways. This unique reactivity originates from its empty p-orbital electron structure, enabling preferential formation of more stable B-C bonds (bond dissociation energy ≈ 4.5 eV) compared to Ti-C bonds (3.86 eV) [112], providing a thermodynamic basis for suppressing excessive TiC formation. B not only competes with Ti for reaction with C thermodynamically but also regulates carbon diffusion kinetics. The controlled addition of B or TiB_2_ enables precise interfacial chemical regulation by effectively suppressing excessive TiC formation while preserving the structural integrity of GNPs and enhancing interfacial bonding strength [113]. Hou et al. [31] utilized low-energy ball milling to uniformly disperse boron powder onto the surface of the TA15 matrix. During the following pre-pressing and hot extrusion processes, B atoms selectively diffused into the vacancy defects of GNPs, forming B–C bonds that filled these structural defects and increased the activation energy required for carbon atom diffusion, thus preventing excessive carbon diffusion. Meanwhile, B atoms adsorbed onto the bridge sites of C–C bonds in GNPs, which expanded the interlayer spacing of the crystal planes. This resulted in the creation of a nanoscale composite interfacial structure containing TiC and TiB phases, as depicted in Figure 33.

Wang et al. [23] conducted a similar study using TiB_2_ particles as the boron source. The TiB_2_ particles and GNPs were co-dispersed into the titanium-based powder. The TiB_2_ reacted with the Ti matrix to form TiB whiskers (TiBw), which were distributed near the interface and obstructed the diffusion path of carbon atoms from GNPs to the Ti matrix. Additionally, because the interfacial phase contained TiBw, when cracks propagated to the TiBw, the whiskers bridged both sides of the crack. Additional energy was required to either pull out or fracture the whiskers from the matrix, resulting in superior plasticity compared to the method used by Hou [31]. Furthermore, differing from Hou’s study [31], the research by Wang et al. [23] revealed that TiBw forms a three-dimensional pinning structure in the interfacial region. This structure synergistically strengthens the interface and physically impedes crack propagation through mechanical interlocking and stress field effects. In contrast, Hou et al. adopted a more direct approach by incorporating elemental boron powder, which primarily focuses on atomic-scale chemical modification and competitive diffusion. From a design standpoint, Hou’s route emphasizes chemical regulation of carbon activity and diffusion, whereas Wang’s [23] route emphasizes microstructural scaffolding (TiBw pinning and crack-bridging); combining both levers is likely the most robust path toward simultaneously strong and damage-tolerant interfaces.

### 4.3. Interface Architecture Engineering Toward Efficient Load Transfer in Graphene-Reinforced Titanium Matrix Composites

Merely optimizing the interfacial structure to suppress interfacial reactions may be insufficient to achieve optimal mechanical properties, owing to the inherent chemical and physical differences between graphene and titanium-based materials [21,69]. An ideal interface should not only exhibit low reactivity but also possess high-strength bonding, efficient load transfer capability, and a simultaneous improvement in both strength and ductility, so as to achieve breakthrough improvements in graphene-reinforced TMCs.

**Figure 34:**
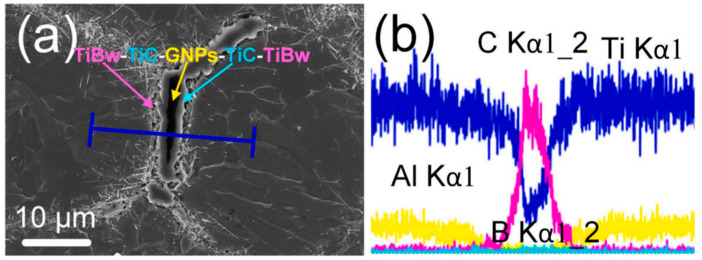
The grain boundaries of composite (**a**), and the corresponding EDS results (**b**) [114].

**Figure 35:**
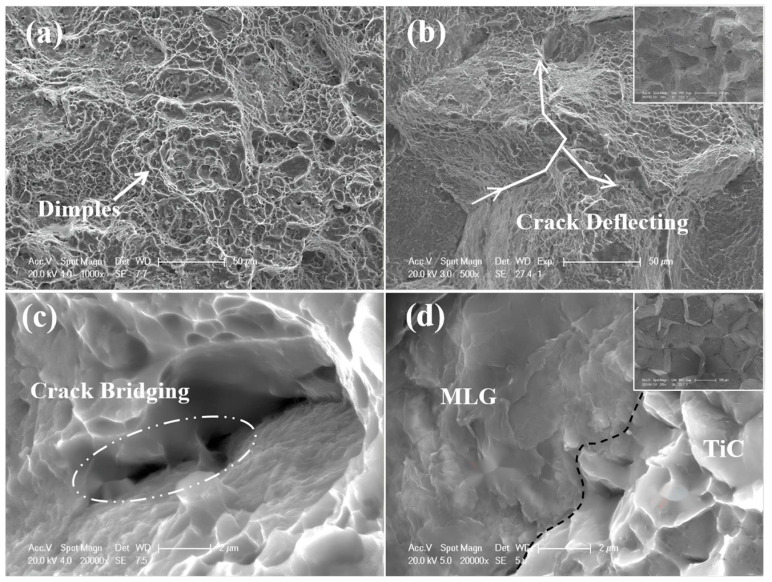
SEM micrographs of tensile fracture surfaces of pure TC4 (**a**), 0.15 wt.% MLG/TC4 composite, showing the crack deflection (**b**) and crack bridging (**c**), 0.25 wt.% MLG/TC4 composite, showing the morphology of MLG embedded on the fractured TiC surface (**d**) [43].

Strategies employing 3D interfacial architectures may offer a promising pathway to achieve strong interfacial bonding and overcome the strength-ductility trade-off in Graphene Nanoplatelet-Reinforced Titanium Matrix Composites (GNPs/TMCs). Ren et al. [114] designed a three-dimensional interfacial architecture consisting of in situ TiBw and a sandwich-like TiC–GNPs–TiC interlayer (Figure 34). TiBw penetrated into adjacent Ti6Al4V grains and mechanically anchored the interlayer, forming a root–soil-like interlocked topology that couples chemical bonding (TiC) with mechanical interlocking (TiBw). This architecture re-routes crack propagation along the TiC–GNP–TiC network boundaries and activates graphene bridging, thereby enhancing energy dissipation during fracture. The key implication is that interface topology can “program” crack paths, rather than merely strengthening a planar interface. Shang et al. [43] fabricated MLG/Ti6Al4V composites with a 3D network structure in which TiC and MLG acted synergistically to reinforce the interfacial regions. Tensile tests revealed that, compared to pure TC4 fracture surfaces (Figure 35a), cracks in the composite with 0.15 wt.% MLG propagated along the boundaries of the discontinuous TiC–MLG network. Unfractured MLG bridged the crack flanks (Figure 35b,c), bearing applied loads and delaying crack opening—leveraging graphene’s ultra-high tensile strength to counteract crack propagation forces, which is far more effective than the passive crack deflection of planar interfaces, thereby effectively inhibiting further crack propagation. These observations demonstrate that well-engineered interfacial microstructures play a decisive role in controlling crack paths and enhancing fracture resistance [115,116].

While the 3D network architecture approach has shown remarkable promise, further innovation is needed to fully harness the potential of graphene reinforcement.

**Figure 36:**
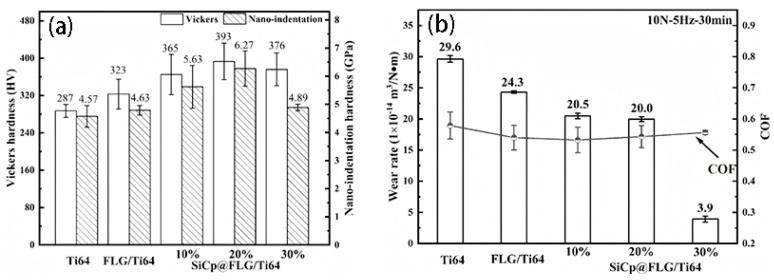
(**a**) Matrix hardness value; (**b**) comparative bar chart for the wear properties [96].

Solution strengthening can complement interfacial architecture by increasing matrix strength and delaying plastic instability, but it must be integrated with interface design rather than treated as a standalone mechanism. For example, Yan et al. [96] achieved an interstitial solid solution of solute atoms such as Si and C in the Ti-6Al-4V matrix and TiC. During the sintering process, SiC nanoparticles react with the titanium matrix according to the reaction formula Ti + SiC → TiC + [Si], where [Si] represents silicon in a solid solution state. When solute atoms like Si enter the interstitial sites of the matrix structure in the form of an interstitial solid solution, they disrupt the original lattice symmetry of the matrix, generating a localized lattice distortion stress field. This strong distortion stress field exerts a prominent pinning effect on dislocations along slip systems, greatly increasing the critical shear stress for dislocation motion. Overcoming this stress field requires additional energy consumption. Solution strengthening significantly enhanced the matrix hardness, as shown in Figure 36a, and directly resulted in a reduced wear rate, as demonstrated in Figure 36b. Yu et al. [35] utilized the substitutional interaction of Co atoms with the pure titanium matrix lattice, which differs from conventional interstitial solid solutions. Since the pure titanium matrix consists solely of α-Ti, its lattice atomic arrangement contains substitutional sites. When Co atoms replace some Ti atoms in the form of a substitutional solid solution, the difference in atomic radii between the two disrupts the original lattice symmetry of the matrix, generating a localized lattice distortion stress field. When external forces are applied to the material, dislocations, which are the primary carriers of plastic deformation, must overcome the stress field in order to continue their movement. On one hand, the lattice distortion increases the frictional resistance to dislocation motion, requiring additional energy consumption. On the other hand, as a β-stabilizing element, Co lowers the β-phase transition temperature of Ti after solid solution, allowing partial retention of β-Ti during cooling and forming a dual-phase α-Ti/β-Ti structure. The presence of α/β phase interfaces further hinders dislocation transmission, indirectly enhancing the solution strengthening effect.

It is important to note that regardless of which element acts as the solute atom, its concentration must be strictly controlled within the solid solution limit. Once exceeding the solid solution limit, excess solute atoms will precipitate to form brittle intermetallic compounds, which easily become stress concentration sources and instead reduce material toughness—thus the solid solution limit is a key threshold for balancing strength and ductility. Solution strengthening in TMCs is governed by lattice distortion-induced dislocation pinning (via interstitial/substitutional solutes), with the key being strict control of the solute concentration within the solid solubility limit to synergistically enhance strength and preserve ductility.

Dual-scale layered architectures overcome the strength–ductility trade-off in conventional uniformly dispersed composites through three mechanisms: physical confinement suppressing interfacial reactions, micro-nano division of labor for synergistic strengthening, and multistage toughening. Feng et al. [97] drew inspiration from the nacre-inspired brick-and-mortar structure to overcome the strength–ductility trade-off in GNP-reinforced TMC via a dual-scale layered architectural design. Low-energy ball mixing preserved the intrinsic structure of GNPs, while rapid hot-press sintering and layered hot rolling were utilized to construct a macroscopic laminated structure consisting of alternating pure Ti6Al4V layers and GNP-reinforced composite layers. Notably, the alternating pure Ti6Al4V layers exploit the excellent plastic deformation capacity of α-Ti/β-Ti dual-phase titanium alloy, acting as ductile buffers to relieve Ti-C interfacial residual stress during loading and impede catastrophic crack propagation—directly enabling the retention of 17.39% elongation alongside enhanced strength. At the microscale, a “sandwich” architecture comprising two TiC layers encapsulating a GNP interlayer was formed. A controlled interfacial TiC reaction layer with a thickness of 20–90 nm achieved strong bonding between the GNPs and the matrix. Furthermore, the layered configuration promoted grain refinement, reducing the average grain size to 1.36 μm and weakening texture intensity. Mechanical tests demonstrated that the composite exhibited a yield strength of 1064 MPa—an increase of 18.13% compared to pure Ti6Al4V—while maintaining an elongation of 17.39%, illustrating a remarkable synergy between strength and ductility.

**Figure 37:**
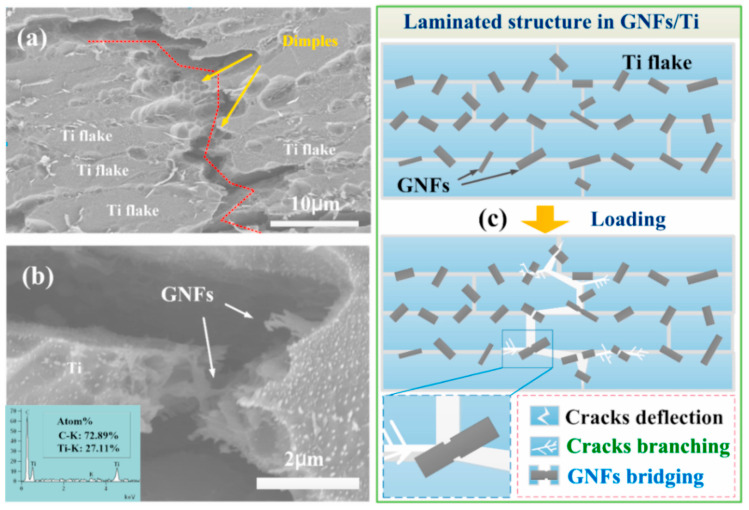
SEM micrographs of the profile microstructure near the fracture surface. (**a**) The tortuous micro-cracks propagated along the fake Ti boundary and (**b**) the presence of the GNF pull-out and bridging state; (**c**) schematic illustration of the fracture mechanism in laminated GNF/Ti composites [108].

**Figure 38:**
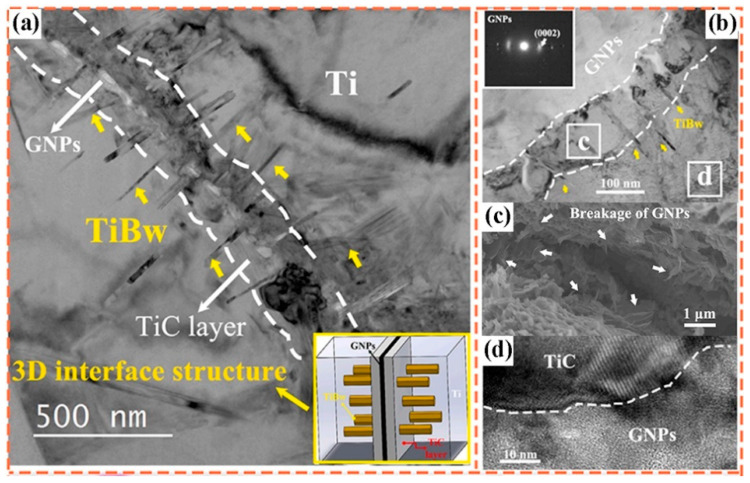
(**a**) TEM images of the interface structure of 3%-TiBw-(GNPs)/Ti composites with an inset of 3D interface configuration diagram, (**b**) highly magnified image of interface region, (**c**) fractured morphologies of specimens after dynamic compression (along CD), (**d**) high-resolution TEM image of TiC-GNP interface characteristics [63].

Mu et al. [108] employed a novel built-in grooved ball milling (BGBM) technique to rapidly transform spherical titanium powder into flaky sheets via a micro-rolling mechanism. This micro-rolling strategy represents an innovation in powder deformation mode, achieving precise control over titanium particle morphology through low-energy, directional plastic deformation. Unlike conventional ball milling, which risks oxidizing titanium powder, the low-energy micro-rolling effect of BGBM reshapes Ti particles while minimizing surface oxidation—critical for preserving Ti matrix integrity—and induces oriented adsorption of GNFs on Ti flake surfaces. This process enables the self-assembly of an innovative brick-and-mortar lamellar microstructure, where Ti flakes act as “bricks” and GNF/TiC interfaces serve as “mortar”. As depicted in Figure 37a, post-compression analysis revealed a zigzag crack propagation along the lamellar boundaries, significantly extending the crack paths and demonstrating enhanced toughening and energy absorption. Simultaneously, GNFs bridged crack flanks depicted in Figure 37b,c, suppressing crack opening through combined pull-out and bridging mechanisms to drastically increase crack-growth resistance while ensuring ductility. The oriented GNFs in the “mortar” layer create directional stress transfer channels aligned with the Ti flake plane, leveraging the anisotropic mechanical behavior of titanium alloys to enable efficient load transmission and avoid local stress concentration—explaining why the Ti-based composite achieves an extraordinary yield strength of 2057 MPa without sacrificing ductility (28% fracture strain). Liu et al. [63] utilized the template effect of GNPs during hot compression to induce the perpendicular growth of TiBw. GNPs’ planar structure provides ideal heterogeneous nucleation sites for TiBw, as their lattice mismatch with TiBw is far lower than with Ti matrix—a key advantage for Ti-based composites—and the compressive stress during hot processing drives TiBw to grow perpendicularly through GNP/TiC layers into the Ti matrix. These TiBw grew through GNPs/TiC layers and into the adjacent titanium matrix, as illustrated in Figure 38a,b, forming a three-dimensional reinforcement network composed of aligned TiBw, TiC layers, and GNPs. The strong bonding between TiC and GNPs, as shown in Figure 38c,d, provided robust interfacial adhesion, effectively inhibiting interfacial debonding. This outstanding performance is attributed to the synergistic effects of reinforcement alignment, texture-induced strengthening, and multiscale coupling among GNPs, TiC, and TiBw.

Despite the impressive strength ductility synergy reported in architected interfaces, two engineering issues are often under-discussed: anisotropy and property scatter. Networked or laminated architectures inevitably introduce direction-dependent deformation and fracture modes, while local variations in connectivity, TiC continuity, and porosity clustering can amplify scatter in strength and fatigue life. Future studies should therefore report standardized interface descriptors and correlate them with not only monotonic tensile properties but also fatigue, creep, and thermal-cycling stability.

In summary, breaking the performance bottleneck of graphene-reinforced TMCs requires shifting from passive reaction suppression to active multifunctional interface architecture design. The most effective designs simultaneously control TiC morphology, build robust load-transfer pathways, and program crack trajectories for energy dissipation. Establishing quantitative links between interface descriptors and in-service reliability will be essential for translating these architectures into engineering components.

## 5. Conclusions and Prospects

Significant advances have been made in the fabrication of graphene-reinforced TMCs, with PM and SPS emerging as the leading techniques due to their excellent control over microstructure and interfacial properties. However, achieving uniform dispersion of graphene and precise regulation of its content remain critical challenges. The difficulty in attaining uniform dispersion primarily stems from the weak interfacial bonding between graphene and the titanium matrix, coupled with graphene’s inherent tendency to agglomerate. Controlling the interfacial reaction is particularly crucial: insufficient reaction leads to low interfacial bonding strength, while excessive reaction results in the formation of brittle TiC phases that compromise ductility. Therefore, the core of future development in this field lies in systematically matching the type of graphene to the target performance and processing requirements, and achieving optimal synergy between material structure, processing, and application through low-cost, controllable preparation and modification technologies. Specifically:

1. Targeted Selection of Reinforcement Phase: The choice of graphene type must be rigorously matched to specific performance objectives. GNPs are the preferred choice when pursuing high-strength, high-modulus, and three-dimensional network reinforcement effects. GNSs or GNFs are more suitable when the focus is on inhibiting crack propagation through their lamellar morphology and enhancing interfacial mechanical interlocking. For systems requiring a balance between reinforcement and plastic accommodation in environments prone to strong interfacial reactions, MLG or even FLG can be selected. In systems with poor dispersion or requiring low-temperature interface regulation, GO, leveraging its surface functional groups, can effectively improve process feasibility and provide potential for subsequent in situ functionalization. Despite their diversity, all types rely on their ultra-high intrinsic mechanical properties and unique two-dimensional topology to provide the core functions of load transfer, crack deflection, and interface strengthening in composites.
2. Strategies for Improving Dispersion Uniformity: To enhance dispersion uniformity, various techniques such as ball milling, ultrasonic dispersion, surface modification, and thermal processing have been widely applied in research. Furthermore, interface design strategies combined with multiscale simulations provide a theoretical foundation for effective dispersion. Cross-scale models reveal the thermodynamic and kinetic mechanisms during the dispersion process, thereby guiding the optimization of dispersion parameters and facilitating the design of structurally stable, homogeneous composites. However, their effectiveness is always constrained by three key factors: dispersion uniformity, interfacial bonding state, and structural integrity.
3. Regulation and Optimization of Interfacial Reactions: Studies have shown that rapid sintering at low temperatures under high pressures can effectively suppress the formation of excessive TiC. When combined with surface modification techniques and the addition of alloying elements, this strategy can further inhibit carbon atom diffusion. Simultaneously, constructing dual-scale layered or 3D network architectures that promote graphene alignment along grain boundaries can synergistically optimize load transfer and crack deflection effects. These strategies collectively enhance the mechanical properties of graphene-reinforced TMCs by concurrently optimizing interfacial integrity and composite toughness.

Future research efforts should prioritize the following directions:

1. Toward a Materials Genomics Approach for Composite Design. Future efforts must move beyond trial-and-error selection of graphene types (GNPs, GNSs, FLG, etc.) and matrix compositions. A more rational approach involves establishing quantitative “interface property maps” that link graphene characteristics (layer number, defect density, functionalization), matrix chemistry, and processing windows to final interfacial structure and mechanical performance. This requires integrating high-throughput computational screening with targeted experimental validation. The goal is to identify forbidden zones in the design space and pinpoint promising compositional niches that promote the formation of thin, discontinuous TiC or other beneficial interphases for enhanced load transfer. The critical challenge lies in accurately modeling and predicting the behavior of multi-component interfaces under non-equilibrium processing conditions.
2. Active Design and Real-time Control of Interface Architecture. The prevailing strategy has been to suppress interfacial reactions. A more ambitious goal is to actively design and control them in situ. This involves pioneering novel interfacial engineering strategies, such as the pre-placement of nanoscale diffusion barriers or catalytic layers with atomic precision, potentially via advanced deposition techniques on powder surfaces. Furthermore, exploring in situ diagnostic tools coupled with rapid process control (adaptive SPS, laser processing) could enable real-time monitoring and feedback during sintering to freeze the interface at a desired state. The key scientific question is whether we can dynamically steer the reaction pathway (Ti + C → TiC) to favor nucleation of isolated nanoparticles over continuous film growth, leveraging insights from non-equilibrium thermodynamics and reaction kinetics.
3. Manufacturing of Architected Composites with Cross-Scale Synergy. The true potential of graphene reinforcement may only be unlocked through deliberate spatial design at multiple scales. Future work should focus on developing hybrid manufacturing routes that combine the microstructural control of PM/SPS with the geometric freedom of additive manufacturing or severe plastic deformation. This would enable the fabrication of functionally graded or topologically optimized components where graphene reinforcements are placed strategically—for instance, concentrated in high-stress regions or aligned along specific load paths. The major hurdles are ensuring graphene survival during high-energy AM processes and achieving robust interfacial bonding in such heterogeneous structures. The vision is to progress from homogeneous composites to composite systems with tailored property profiles.
4. AI and Machine Learning in Material Optimization. Machine learning and artificial intelligence demonstrate tremendous potential to revolutionize traditional materials development processes, but their application must be grounded in an understanding of the limitations of current models and the unique challenges of TMCs. Future research should focus on the following key areas: First, current literature is highly fragmented, and key descriptions are often missing; efforts should be made to establish a comprehensive, rigorously curated open-source database that includes process parameters, microstructural features, and mechanical properties. Second, the development of hybrid intelligent models that integrate physical laws is crucial. Pure data-driven models function like a “black box” and have limited extrapolation capabilities. The next generation of models must incorporate physical principles and domain knowledge (e.g., Hall–Petch effect) to enhance interpretability and improve prediction accuracy for unknown components or processes. Finally, advancing active learning and autonomous laboratories is essential. Beyond prediction, machine learning can guide experimental design. By establishing a Bayesian optimization loop, it will efficiently explore the vast process parameter space (e.g., graphene content, ball-milling time, sintering temperature/time) and automatically recommend the next set of experiments with the most informative potential, approaching multi-objective optimization.

## Figures and Tables

**Table 1:** The detailed Raman spectrum results of the ball-milled composite powders [24].

Ball-Milling Time	D Band	G Band	2D Band	ID/IG
GNPs	1362.69	1586.29	2723.45	0.26
2h	1350.62	1582.31	2702.70	0.56
5h	1347.44	1583.86	2693.27	1.26
10h	1350.62	1590.03	2682.48	1.52
15h	1331.54	1577.68	2677.08	1.67

**Table 2:** The influence of key parameters of different processes.

Process Link	Process Parameters	Influence Mechanism	Reference
Raw Material Pretreatment	Graphene Content	Excessive graphene content promotes agglomeration and elevates material porosity, wherein interconnected agglomerates and pores nucleate structural defects.	[25,26,27]
Powder Mixing	Ball-Milling Time	Increasing ball-milling time facilitates the dispersion of graphene, while simultaneously inducing cold welding between graphene and titanium powder and elevating oxygen content.	[23,26,28,29]
	Rotational Speed	Low-Energy Ball Milling: Only achieves physical adhesion of graphene, which easily forms micron-sized agglomerates on the surface of titanium powder. High-Energy Ball Milling: Graphene can be dispersed into nanoscale particles, but strict control of processing time and the addition of stearic acid as a protective agent are required. Otherwise, excessive shear will damage the sp^2^ structure of graphene.	[24,30,31,32]
	Process Control Agent	To prevent powder over-cold welding, minimize grinding jar/ball contamination, suppress temperature rise, and facilitate homogeneous graphene dispersion.	[33,34]
Compaction	Compaction Pressure	Higher compaction pressure significantly increases green density and reduces porosity.	[35]
Sintering Process	Sintering Temperature	Elevated sintering temperatures enhance material densification, but simultaneously intensify interfacial reactions, leading to excessive TiC formation.	[27,31,36,37]
	Holding Time	Prolonged holding time enhances material densification and promotes grain growth but inevitably causes significant coarsening of pre-existing TiC particulates.	[27,38]
	Sintering Atmosphere	Excessive air exposure promotes significant material oxidation, whereas ultra-high-purity inert gas shielding coupled with a moderate vacuum represents the optimal sintering atmosphere strategy.	[25,39]

**Table 3:** Fabrication methods and performance of graphene-reinforced titanium matrix composites.

Base Matrix	Type of Filler	Its Concentration	Mixing Method	Composite Fabrication Method	Results Obtained	Reference
Ti64	GNSs	0.5 wt.%	Mechanical rocking mixing	SLM	Tensile Strength: 1526 MPa; Young’s Modulus: 145 GPa	[13]
TC4	GNPs + TiB_2_	0.1wt.% GNPs 0.05 wt.% TiB_2_	Ball milling	Fast Hot-Press Sintering	Tensile strength: 1207 MPa Elongation: 2.3%	[23]
TC4	GNPs	0.3 wt.%	High-energy ball milling	SPS	Yield strength: 1482 MPa Compressive strength: 1929 MPa Hardness: 539 HV	[24]
CP-Ti	GNSs	1 wt.%	Ball milling	-	Shear yield strength: 728 MPa Ultimate shear strength: 754 MPa Microhardness: 613 HV	[25]
TC21	GNPs	0.3 wt.%	Ball milling	SPS	Tensile strength: 1167 MPa Yield strength: 1041 MPa Elongation: 4.4%	[37]
TC4	Ni-P@GNFs	1 wt.%	Ball milling	HIP	Yield strength: 1299.59 MPa Microhardness: 410.10 HV Elongation: 25.76%	[61]
Ti662	Gn	0.5%	Ball milling	Microwave sintering	Microhardness: 514.32 HV	[65]

**Table 4:** Comparison of fabrication methods for graphene-reinforced titanium matrix composites.

Fabrication Method	Advantages	Disadvantages	Typical Performance Outcomes	References
Powder Metallurgy	1. High material utilization, near-net shape forming and good microstructural homogeneity. 2. Scalable for bulk production, supports custom composite powder design.	1. Prolonged milling time may lead to excessive graphene damage. 2. High-energy input can cause contamination and oxidation.	Enhanced strength and hardness, but ductility can be compromised by poor dispersion or thick TiC layers.	[40]
Spark Plasma Sintering	1. Achieves high-density sintering in a short time. 2. Minimizes porosity and ensures high material densification. 3 Effectively inhibits grain growth via short-time sintering.	1. Limited scalability for mass production. 2. Difficulty in controlling TiC formation at higher sintering temperatures.	Excellent strength and hardness due to fine grains and high density; balance with ductility possible with optimal parameters.	[66,67]
Selective Laser Melting	1. Rapid manufacturing of complex geometries. 2. High precision and fine microstructure. 3 Achieves near-net shaping with minimal waste	1. Limited material compatibility. 2. High cost and slow production speed for large components. 3. Prone to residual stresses	Very high tensile strength reported, but ductility often low due to defects and microstructure.	[13]
Hot Isostatic Pressing	1. Excellent defect closure effect, enhancing material densification and uniformity. 2. Suitable for large-scale production.	1. Long sintering time and high energy consumption. 2. May agglomerate during the high-temperature process.	High fatigue strength and creep resistance, but may have lower room-temperature toughness due to TiC coarsening.	[68]
Microwave Sintering	1. Lower sintering temperature, energy-efficient and short processing time. 2. Ensures temp uniformity, prevents local overheating damage	1. Immature technology, only for small-scale experiments 2. Only suitable for specific materials (e.g., small-sized particles, thin-film materials).	Good enhancement in hardness and wear resistance; reported densities > 98%.	[69]

**Table 5:** Processing parameters for 0.25 wt.% GNP-Ti composites [79].

Sample Batch	Composite Constituents Mixing					Consolidation				
	Method	Medium	Charge Ratio	Speed	Time	Compaction Pressure	Sintering Temperature	Sintering Time	Heating and Cooling Rate	Sintering Environment
1	Dry ball milling	Tungsten carbide balls	Balls to powder ratio 6:1	225 rpm	3 h	400 MPa	1100 °C	2 h	10 °C/min	10^−3^ vacuum
2	Wet ball milling	Tungsten carbide balls	Balls to powder ratio 6:1	225 rpm	3 h					
3	Rotator mixing	Stainless steel blade	Volume filled 1/3	300 rpm	3 h					

**Table 6:** Comparison of primary densification techniques.

Method	Advantages	Disadvantages
SPS	1. Achieves rapid densification and high density in a short time; 2. Prevents grain coarsening through pulsed current and isostatic pressure; 3. Can complete sintering at relatively lower temperatures.	1. Requires advanced equipment and is relatively costly; 2. Sensitive temperature control may lead to excessive TiC formation; 3. Limited capability for processing components with complex geometries.
HIP	1. Provides excellent densification, effectively eliminating pores and defects; 2. Suitable for large-scale production and capable of processing components with complex shapes; 3. Contributes to improved high-temperature performance.	1. Longer sintering times and higher temperatures are required; 2. May lead to excessive TiC formation, affecting the mechanical properties of the material.
Microwave Sintering	1. Significantly reduces sintering temperature and time; 2. Suppresses excessive TiC formation and mitigates over-reaction; 3. Enhances material hardness and wear resistance.	1. Control over graphene dispersion and excessive reaction is relatively complex; 2. Requires specialized equipment and has limited applicability.
SLM	1. Enables rapid solidification, reducing excessive TiC formation; 2. Creates refined microstructures, enhancing mechanical properties; 3. Suitable for producing components with complex geometries.	1. May cause partial structural damage to graphene due to material overheating; 2. Challenges in controlling reinforcement size may lead to non-uniform mechanical properties.

## Data Availability

No new data were created or analyzed in this study. Data sharing is not applicable to this article.

## Appendix A

**Abbreviation**	**Full Name**	**Description**
Ti	Titanium	It is the chemical symbol for titanium.
TMCs	Titanium Matrix Composites	Composites made of a titanium alloy matrix and reinforcing phase(s), typically in the micrometer to nanometer range.
TC4/Ti64	Ti-6Al-4V	A titanium alloy matrix consisting of titanium (90%), aluminum (6%), and vanadium (4%), on the micrometer scale.
GNPs	Graphene Nanoplatelets	Thin graphene sheets consisting of a few layers (typically 1–10 layers, 1–10 nm thick).
GNSs	Graphene Nanosheet	Single or few-layered graphene with 1 nm thickness for single-layer graphene.
GR	Graphene	In this paper, the term is used as a general reference to graphene-based materials.
GNFs	Graphene Nanoflakes	Flake-like graphene with multiple layers (typically 2–10 layers, 1–10 nm thick).
CP-Ti	Commercially Pure Titanium	Titanium with minimal alloying elements, typically over 99% pure titanium, often in the micrometer scale.
MLG	Multi-Layer Graphene	Graphene made up of multiple stacked layers (typically 2–10 layers, 1–3 nm thick per layer). Properties are between single-layer graphene and bulk graphite.
FLG	Few-Layered Graphene	Graphene consisting of a few layers, typically 2–10 layers, with thickness between 1–10 nm.
GO	Graphene Oxide	Oxidized graphene, typically with functional groups (e.g., hydroxyl, carboxyl) attached to the surface, typically on a nanometer scale (~1–2 nm thickness).
CNTs	Carbon Nanotubes	One-dimensional tubular carbon nanomaterials, referring to cylindrical carbon nanostructures with a tubular atomic arrangement of carbon atoms, typically with a diameter of 1–100 nm and a length ranging from micrometers to centimeters.
HCP	Hexagonal Close-Packed	A crystalline structure with atoms arranged in a hexagonal pattern.
BCC	Body-Centered Cubic	A crystal structure where atoms are arranged in a body-centered cubic lattice.
BN	Boron Nitride	-
PM	Powder Metallurgy	-
MA	Mechanical Alloying	-
SPS	Spark Plasma Sintering	-
BPR	Ball-to-Powder Ratio	-
PCA	Process Control Agent	-
HIP	Hot Isostatic Pressing	-
SLM	Selective Laser Melting	-
RHR	Reactive Hot Rolling	-
BGBM	Built-In Grooved Ball Milling	-

## References

[1] Hayat M.D., Singh H., He Z., Cao P. (2019). Titanium metal matrix composites: An overview. Compos. Part A Appl. Sci. Manuf..

[2] Md Ali A., Omar M.Z., Hashim H., Salleh M.S., Mohamed I.F. (2021). Recent development in graphene-reinforced aluminium matrix composite: A review. Rev. Adv. Mater. Sci..

[3] Wang S., Wang J., Xu Z., Wang J., Li R. (2024). Numerical calculation of overlapping line heating for marine titanium alloy curved plate. Ocean Eng..

[4] Dubey S., Soboyejo W.O., Srivatsan T.S. (1997). Deformation and Fracture Properties of Damage Tolerant In-situ Titanium Matrix Composites. Appl. Compos. Mater..

[5] Xue N.P., Wu Q., Zhang Y., Li B.H., Zhang Y.D., Yang S., Zhu Y., Guo J., Gao H.J. (2022). Review on research progress and comparison of different residual stress strengthening methods for titanium alloys. Eng. Fail. Anal..

[6] Papageorgiou D.G., Kinloch I.A., Young R.J. (2017). Mechanical properties of graphene and graphene-based nanocomposites. Prog. Mater. Sci..

[7] Razaq A., Bibi F., Zheng X., Papadakis R., Jafri S.H.M., Li H. (2022). Review on graphene-, graphene oxide-, reduced graphene oxide-based flexible composites: From fabrication to applications. Materials.

[8] Shchegolkov A.V., Shchegolkov A.V., Kaminskii V.V. (2026). Carbon Nanotubes and Graphene in Polymer Composites for Strain Sensors: Synthesis, Functionalization, and Application. J. Compos. Sci..

[9] Lee C., Wei X., Kysar J.W., Hone J. (2008). Measurement of the Elastic Properties and Intrinsic Strength of Monolayer Graphene. Science.

[10] Han X., Wang R., Kang P., Li W., Wu G. (2024). First-Principles Study on the Influence of Crystal Structures on the Interface Properties of Graphene/Titanium Composites. Coatings.

[11] Lee H., Lordejani A.A., van Goor L., Jurov A., Koutsioukis A., Ruan S., Santhosh N.M., Zarei F., Barreneche C., Cvelbar U. (2025). Review on properties; physics, and fabrication of two-dimensional material-based metal-matrix composites (2DMMCs) for heat transfer systems. Renew. Sustain. Energy Rev..

[12] Wen X., Joshi R. (2020). 2D materials-based metal matrix composites. J. Phys. D Appl. Phys..

[13] Yan Q., Chen B., Li J.S. (2021). Super-high-strength graphene/titanium composites fabricated by selective laser melting. Carbon.

[14] Mutuk T., Gürbüz M. (2021). Si_3_N_4_/Graphene binary particles reinforced hybrid titanium composites and their characterization. Int. J. Mater. Res..

[15] Cao H.-C., Liang Y.-L. (2020). The microstructures and mechanical properties of graphene-reinforced titanium matrix composites. J. Alloys Compd..

[16] Gürbüz M., Mutuk T., Uyan P. (2020). Mechanical, Wear and Thermal Behaviors of Graphene Reinforced Titanium Composites. Met. Mater. Int..

[17] Chen D., Li J., Sun K., Fan J. (2023). Graphene-reinforced metal matrix composites: Fabrication, properties, and challenges. Int. J. Adv. Manuf. Technol..

[18] Yoganandam K., Mohanavel V., Vairamuthu J., Kannadhasan V. (2020). Mechanical properties of titanium matrix composites fabricated via powder metallurgy method. Mater. Today Proc..

[19] Gao Y., Zou J., Wang H., Han Y. (2023). Interfacial reaction and interfacial strengthening mechanism of graphene nanosheets reinforced powder metallurgy nickel-based superalloy composite. Mater. Charact..

[20] Suryanarayana C. (2019). Mechanical Alloying: A Novel Technique to Synthesize Advanced Materials. Research.

[21] Sharma D., Singla V.K., Singh S. (2022). Effect of multi-layer graphene on microstructure and mechanical properties of titanium-based composites. Proc. Inst. Mech. Eng. Part C J. Mech. Eng. Sci..

[22] Alsalama M., Hamoudi H., Youssef K.M. (2021). The effect of graphene structural integrity on the power factor of tin selenide nanocomposite. J. Alloys Compd..

[23] Wang H., Zhang H.M., Cheng X.W., Chang S., Mu X.N. (2022). Effect of ball milling time on microstructure and mechanical properties of graphene nanoplates and TiBw reinforced Ti–6Al–4V alloy composites. Mater. Sci. Eng. A.

[24] Zhou Y., Dong L., Yang Q., Huo W., Fu Y., Yu J., Liu Y., Zhang Y. (2021). Controlled Interfacial Reactions and Superior Mechanical Properties of High Energy Ball Milled/Spark Plasma Sintered Ti–6Al–4V–Graphene Composite. Adv. Eng. Mater..

[25] Haghighi M., Shaeri M.H., Sedghi A., Djavanroodi F. (2018). Effect of Graphene Nanosheets Content on Microstructure and Mechanical Properties of Titanium Matrix Composite Produced by Cold Pressing and Sintering. Nanomaterials.

[26] Zhang J., Min B.W., Gu H., Wu G.Q., Dai G.Q., Sun Z.G. (2024). Grain Refinement and Mechanical Enhancement of Titanium Matrix Composites with Nickel-Coated Graphene Nanoflakes: Influence of Particle-Size Mismatch. Crystals.

[27] Gürbüz M., Mutuk T. (2017). Effect of process parameters on hardness and microstructure of graphene reinforced titanium composites. J. Compos. Mater..

[28] Dong L., Zhang W., Fu Y., Lu J., Liu X., Tian N., Zhang Y. (2021). Reduced Graphene Oxide Nanosheets Decorated with Copper and Silver Nanoparticles for Achieving Superior Strength and Ductility in Titanium Composites. ACS Appl. Mater. Interfaces.

[29] Pekok M., Setchi R., Ryan M., Han Q. (2021). Effect of Milling Speed and Time on Graphene-Reinforced AA2024 Powder. Sustainable Design and Manufacturing 2020.

[30] Zhu J., Yuan M., Pei X., Zhou X., Li M. (2024). The Effect of Stearic Acid on Microstructure and Properties of (Ti_2_AlC + Al_2_O_3_)p/TiAl Composites. Metals.

[31] Hou J., Chi F., Chi L., Cui G., Chen W., Zhang W. (2021). Effects of the interface and mechanical properties of GNPs/TA15 composites through adding B powders. J. Alloys Compd..

[32] Kozlík J., Stráský J., Harcuba P., Ibragimov I., Chráska T., Janeček M. (2018). Cryogenic Milling of Titanium Powder. Metals.

[33] Ge Y.X., Zhang H.M., Cheng X.W., Fan Q.B., Zhang Z.H., Mu X.N., Liu L. (2021). Towards high performance in Ti-based composite through manipulating nickel coatings on graphene reinforcement. J. Alloys Compd..

[34] Lin K., Fang Y., Gu D., Ge Q., Zhuang J., Xi L. (2021). Selective laser melting of graphene reinforced titanium matrix composites: Powder preparation and its formability. Adv. Powder Technol..

[35] Yu J., Zhao Y., Zhao Q., Zhang W., Huo W., Zhang Y. (2022). Microstructure and Properties of Titanium Matrix Composites Synergistically Reinforced by Graphene Oxide and Alloying Elements. Adv. Eng. Mater..

[36] Wang W., Zhou H., Wang Q., Wei B., Xin S., Gao Y. (2020). Microstructural Evolution and Mechanical Properties of Graphene-Reinforced Ti-6Al-4V Composites Synthesized via Spark Plasma Sintering. Metals.

[37] Yu J., Zhao Q., Huang S., Zhao Y., Zhou Y., Lu J., Dong L., Zhang Y. (2021). Effect of sintering temperature on microstructure and properties of graphene nanoplatelets reinforced TC21 composites prepared by spark plasma sintering. J. Alloys Compd..

[38] Zhang Z.Y., Liang Y.L., Cao H.C., Zhu Y. (2019). The Preparation and Mechanical Properties of a Pure Titanium-Based Matrix Composite Reinforced with Graphene Nanoplatelets. Sci. Adv. Mater..

[39] Liu J., Hu N., Liu X., Liu Y., Lv X., Wei L., Zheng S. (2019). Microstructure and Mechanical Properties of Graphene Oxide-Reinforced Titanium Matrix Composites Synthesized by Hot-Pressed Sintering. Nanoscale Res. Lett..

[40] Mu X.N., Cai H.N., Zhang H.M., Fan Q.B., Wang F.C., Zhang Z.H., Ge Y.X., Shi R., Wu Y., Wang Z. (2018). Uniform dispersion and interface analysis of nickel coated graphene nanoflakes/ pure titanium matrix composites. Carbon.

[41] Ge Y.X., Zhang H.M., Cheng X.W., Fan Q.B., Zhang Z.H., Mu X.N., Liu L., Liu Y.N., Wang B. (2021). Interface evolution and mechanical properties of nickel coated graphene nanoflakes/pure titanium matrix composites. J. Alloys Compd..

[42] Zhang B., Zhang F., Saba F., Shang C. (2021). Graphene-TiC hybrid reinforced titanium matrix composites with 3D network architecture: Fabrication, microstructure and mechanical properties. J. Alloys Compd..

[43] Shang C., Zhang F., Zhang B., Chen F. (2020). Interface microstructure and strengthening mechanisms of multilayer graphene reinforced titanium alloy matrix nanocomposites with network architectures. Mater. Des..

[44] Li Z., Xing S., Wu S., Hou J., Wu S. (2024). A Review on the Interface Structure and Control Between Graphene Nanoplatelets (GNPs) and Ti Matrix of GNPs/Ti Matrix Composites. Metals.

[45] Yan Q., Chen B., Ye W., Zhang T., Wan J., Zhou Q., Shen J., Li J., Lu W.F., Wang H. (2023). Simultaneously improving mechanical; thermal, and anti-wear properties of Ti alloys using 3D-networked graphene as reinforcement. Carbon.

[46] Liu J., Wu M., Yang Y., Yang G., Yan H., Jiang K. (2018). Preparation and mechanical performance of graphene platelet reinforced titanium nanocomposites for high temperature applications. J. Alloys Compd..

[47] Yang Y., Feng C., Zhou Y., Zha X., Bao R., Ke K., Yang M., Tan C., Yang W. (2020). Achieving improved electromagnetic interference shielding performance and balanced mechanical properties in polyketone nanocomposites via a composite MWCNTs carrier. Compos. Part A Appl. Sci. Manuf..

[48] Suryanarayana C. (2022). Mechanical alloying: A critical review. Mater. Res. Lett..

[49] Duan H., Li X., Zhang H., Cheng X., Mu X., Zheng K. (2024). The High-Strain-Rate Impacts Behaviors of Bilayer TC4-(GNPs/TC4) Composites with a Hierarchical Microstructure. Materials.

[50] Huang L.J., Geng L., Peng H.X. (2015). Microstructurally inhomogeneous composites: Is a homogeneous reinforcement distribution optimal?. Prog. Mater. Sci..

[51] Oguntuyi S.D., Johnson O.T., Shongwe M.B. (2021). Spark plasma sintering of ceramic matrix composite of TiC: Microstructure, densification, and mechanical properties: A review. Int. J. Adv. Manuf. Technol..

[52] Rominiyi A.L., Shongwe M.B., Maledi N., Babalola B.J., Olubambi P.A. (2019). Synthesis, microstructural and phase evolution in Ti–2Ni and Ti–10Ni binary alloys consolidated by spark plasma sintering technique. Int. J. Adv. Manuf. Technol..

[53] Falodun O.E., Oke S.R., Obadele B.A., Okoro A.M., Olubambi P.A. (2019). Influence of SiAlON Ceramic Reinforcement on Ti6Al4V Alloy Matrix via Spark Plasma Sintering Technique. Met. Mater. Int..

[54] Singh N., Ummethala R., Karamched P.S., Sokkalingam R., Gopal V., Manivasagam G., Prashanth K.G. (2021). Spark plasma sintering of Ti6Al4V metal matrix composites: Microstructure, mechanical and corrosion properties. J. Alloys Compd..

[55] Wang Q., Zhang Z.H., Liu L.J., Jia X.T., He Y.Y., Sun Y.H., Cheng X.W. (2023). Fabrication of TiB-reinforced titanium matrix composite via spark plasma sintering and vertical bidirectional reactive hot rolling. Mater. Charact..

[56] Yu J., Zhao Y., Zhang W., Zhao Q., Lu J., Huo W., Zhang Y. (2022). A novel heterogeneous network structure titanium matrix composite with a combination of strength and ductility. Mater. Sci. Eng. A.

[57] Zhang X., Alduma A.I.A., Zhan F., Zhang W., Ren J., Lu X. (2025). Effect of Grain Size on Mechanical Properties and Deformation Mechanism of Nano-Polycrystalline Pure Ti Simulated by Molecular Dynamics. Metals.

[58] El-Hadad S., Elsayed A., Shi B., Attia H. (2023). Experimental Investigation on Machinability of α/β Titanium Alloys with Different Microstructures. Materials.

[59] Lei C., Du Y., Zhu M., Huo W., Wu H., Zhang Y. (2021). Microstructure and mechanical properties of in situ TiC/Ti composites with a laminated structure synthesized by spark plasma sintering. Mater. Sci. Eng. A.

[60] Smetanina K.E., Andreev P.V., Nokhrin A.V., Lantsev E.A., Chuvildeev V.N. (2023). Carbon contamination during spark plasma sintering of powder materials: A brief overview. J. Alloys Compd..

[61] Cao X., Ma H., Jia G., Dai G., Guo Y., Sun Z., Liu H., Chang H. (2023). Role of Powder Metallurgical Processing on Mechanical Response of Nickel–Phosphorus-Coated Graphene Nanoflakes/Titanium Matrix Composites. Adv. Eng. Mater..

[62] Cai C., Gao X., Teng Q., Kiran R., Liu J., Wei Q., Shi Y. (2020). Hot isostatic pressing of a near α-Ti alloy: Temperature optimization, microstructural evolution and mechanical performance evaluation. Mater. Sci. Eng. A.

[63] Liu L., Li Y., Zhang H., Cheng X., Mu X., Ge Y. (2021). Breaking through the dynamic strength-ductility trade-off in TiB reinforced Ti composites by incorporation of graphene nanoplatelets. Compos. Part B Eng..

[64] Otte J.A., Zou J., Dargusch M.S. (2022). High strength and ductility of titanium matrix composites by nanoscale design in selective laser melting. J. Mater. Sci. Technol..

[65] Akhil U.V., Radhika N., Ramkumar T., Pramanik A. (2024). Effect of graphene on the tribological behavior of Ti6Al6V2Sn/Gn composite produced via microwave sintering. Int. J. Lightweight Mater. Manuf..

[66] Zhou H., Su Y., Liu N., Kong F., Wang X., Zhang X., Chen Y. (2018). Modification of microstructure and properties of Ti-47Al-2Cr-4Nb-0.3W alloys fabricated by SPS with trace multilayer graphene addition. Mater. Charact..

[67] Yan Q., Chen B., Cao L., Liu K.Y., Li S., Jia L., Kondoh K., Li J.S. (2021). Improved mechanical properties in titanium matrix composites reinforced with quasi-continuously networked graphene nanosheets and in-situ formed carbides. J. Mater. Sci. Technol..

[68] Chen H., Mi G., Li P., Cao C. (2022). Excellent high-temperature strength and ductility of graphene oxide reinforced high-temperature titanium alloy matrix composite fabricated by hot isostatic pressing and heat treatment. Compos. Commun..

[69] Yang W.Z., Huang W.M., Wang Z.F., Shang F.J., Huang W., Zhang B.Y. (2016). Thermal and Mechanical Properties of Graphene–Titanium Composites Synthesized by Microwave Sintering. Acta Metall. Sin. Engl. Lett..

[70] Sharma H., Arora G., Singh M.K., Ayyappan V., Bhowmik P., Rangappa S.M., Siengchin S. (2025). Review of machine learning approaches for predicting mechanical behavior of composite materials. Discov. Appl. Sci..

[71] Dev B., Rahman M.A., Islam M.J., Rahman M.Z., Zhu D. (2023). Properties prediction of composites based on machine learning models: A focus on statistical index approaches. Mater. Today Commun..

[72] Wu X., Zhou Y., Zhang J., Liang J. (2023). Data driven performance prediction of titanium-based matrix composites. Alex. Eng. J..

[73] Nasr M.M., Anwar S.M., Al-Samhan A., Ghaleb M., Dabwan A. (2020). Milling of Graphene Reinforced Ti6Al4V Nanocomposites: An Artificial Intelligence Based Industry 4.0 Approach. Materials.

[74] Pashmforoush F. (2022). Mechanical properties prediction of various graphene reinforced nanocomposites using transfer learning-based deep neural network. Proc. Inst. Mech. Eng. Part E J. Process Mech. Eng..

[75] Malashin I.P., Martysyuk D., Nelyub V., Borodulin A., Gantimurov A., Tynchenko V. (2025). A review of physics-informed and data-driven approaches for manufacturing process optimization in polymer matrix composites. Adv. Manuf. Polym. Compos. Sci..

[76] Ribeiro D.S., Santos J.C., Grieger S., Campos J.L.E., Machado L.R., Pacheco F.G., Fernandes T.F., Haase C.C., Silva D.L., Guterres M. (2023). Measuring the Surface Area Concentration and Specific Surface Area of Mass-Produced Graphene Nanoflakes via Fluorescence Quenching. ACS Appl. Nano Mater..

[77] Mazaheri M., Payandehpeyman J., Hedayatian M. (2023). Agglomeration and interphase-influenced effective elastic properties of Metal/Graphene nanocomposites: A developed mean-field model. Compos. Struct..

[78] Munir K.S., Zheng Y., Zhang D., Lin J., Li Y., Wen C. (2017). Microstructure and mechanical properties of carbon nanotubes reinforced titanium matrix composites fabricated via spark plasma sintering. Mater. Sci. Eng. A.

[79] Mahmood S., Iqbal A., Wadood A., Mateen A., Amin M., Yahia I.S., Zahran H.Y. (2022). Influence of Homogenizing Methodology on Mechanical and Tribological Performance of Powder Metallurgy Processed Titanium Composites Reinforced by Graphene Nanoplatelets. Molecules.

[80] Mu X.N., Cai H.N., Zhang H.M., Fan Q.B., Wang F.C., Zhang Z.H., Wu Y., Ge Y.X., Chang S., Shi R. (2018). Uniform dispersion of multi-layer graphene reinforced pure titanium matrix composites via flake powder metallurgy. Mater. Sci. Eng. A.

[81] Zhang W., Zhou S., Ren W., Yang Y., Shi L., Zhou Q., Liu M. (2021). Uniformly dispersing GNPs for fabricating graphene-reinforced pure Ti matrix composites with enhanced strength and ductility. J. Alloys Compd..

[82] Zhou Q., Liu M., Zhang W., Zhang Z., Sun Y., Ren W., Wei J., Wu P., Ma S. (2024). The distribution of reinforcements in titanium matrix composites enhanced with graphene: From dispersed to networked. Carbon.

[83] Zhang W., Wu P., Wei J., Zhou Q., Liu M. (2024). Simple, low-cost and high-quality fabrication of GNPs@Ti6Al4V powders for high-performance composites Inspired by pearl polishing. Mater. Lett..

[84] Mu X.N., Zhang H.M., Cai H.N., Fan Q.B., Zhang Z.H., Wu Y., Fu Z.J., Yu D.H. (2017). Microstructure evolution and superior tensile properties of low content graphene nanoplatelets reinforced pure Ti matrix composites. Mater. Sci. Eng. A.

[85] Cao Z., Li J.L., Zhang H.P., Li W.B., Wang X.D. (2020). Mechanical and tribological properties of graphene nanoplatelets-reinforced titanium composites fabricated by powder metallurgy. J. Iron Steel Res. Int..

[86] Wei L., Liu X., Gao Y., Lv X., Hu N., Chen M. (2021). Synergistic strengthening effect of titanium matrix composites reinforced by graphene oxide and carbon nanotubes. Mater. Des..

[87] Hu Z., Tong G., Nian Q., Xu R., Saei M., Chen F., Chen C., Zhang M., Guo H., Xu J. (2016). Laser sintered single layer graphene oxide reinforced titanium matrix nanocomposites. Compos. Part B Eng..

[88] Jeje S.O., Shongwe M.B., Rominiyi A.L., Olubambi P.A. (2021). Spark plasma sintering of titanium matrix composite—A review. Int. J. Adv. Manuf. Technol..

[89] Song Y., Liu W., Sun Y., Guan S., Chen Y. (2021). Microstructural Evolution and Mechanical Properties of Graphene Oxide-Reinforced Ti6Al4V Matrix Composite Fabricated Using Spark Plasma Sintering. Nanomaterials.

[90] Liu L., Li Y., Zhang H., Cheng X., Fan Q., Mu X., Guo S. (2021). Good strength-plasticity compatibility in graphene nanoplatelets/Ti composites by strengthening the interface bonding via in-situ formed TiB whisker. Ceram. Int..

[91] Hu Z., Chen F., Xu J., Ma Z., Guo H., Chen C., Nian Q., Wang X., Zhang M. (2018). Fabricating graphene-titanium composites by laser sintering PVA bonding graphene titanium coating: Microstructure and mechanical properties. Compos. Part B Eng..

[92] Akhil U.V., Radhika N., Ramkumar T. (2024). Corrosion behavior of microwave-sintered Ti6Al6V2Sn reinforced with graphene nanoparticles. J. Bio TriboCorros..

[93] Liang A., Jiang X., Hong X., Jiang Y., Shao Z., Zhu D. (2018). Recent Developments Concerning the Dispersion Methods and Mechanisms of Graphene. Coatings.

[94] Skrypnychuk V., Boulanger N., Nordenstrom A., Talyzin A. (2020). Aqueous Activated Graphene Dispersions for Deposition of High-Surface Area Supercapacitor Electrodes. J. Phys. Chem. Lett..

[95] Sainz-Urruela C., Vera-López S., San Andrés M.P., Díez-Pascual A.M. (2022). Surface functionalization of graphene oxide with tannic acid: Covalent vs non-covalent approaches. J. Mol. Liq..

[96] Yan Q., Chen B., Ye W., Wan J., Zhang T., Kou H., Zhou Q., Lu W., Wang H., Shen J. (2022). Extraordinary Antiwear Properties of Graphene-Reinforced Ti Composites Induced by Interfacial Decoration. ACS Appl. Mater. Interfaces.

[97] Feng K., Zhang H., Cheng X., Fan Q., Mu X., Xiong N., Wang H., Duan H. (2023). Breaking through the strength-ductility trade-off in graphene nanoplatelets reinforced titanium matrix composites via two-scale laminated architecture design. Mater. Charact..

[98] Liu L., Zhang H., Cheng X., Mu X., Fan Q. (2022). Graphene nanoplatelets induced laminated heterogeneous structural titanium matrix composites with superior mechanical properties. Ceram. Int..

[99] Zhang Z., Liang Y. (2020). Interfacial mechanical properties of graphene/titanium in composites based on beam search algorithm. Asia Pac. J. Chem. Eng..

[100] Gao T., He H., Liu Y., Bian Z., Chen Q., Xie Q., Liang Y., Xiao Q. (2023). Molecular dynamics simulation of dislocation network formation and tensile properties of graphene/TiAl-layered composites. Surf. Interfaces.

[101] Fonseca A.F., Liang T., Zhang D., Choudhary K., Phillpot S.R., Sinnott S.B. (2018). Titanium-Carbide Formation at Defective Curved Graphene-Titanium Interfaces. MRS Adv..

[102] Hou J., Zhang W., Cui G., Chen W., Ma Q., Wu S. (2022). The graphene nanoplatelets evolution and interface reaction of graphene nanoplatelets/TA15 composites by pre-sintering and hot extrusion. Vacuum.

[103] Kvashina T., Uvarov N., Ukhina A. (2020). Synthesis of Titanium Carbide by Means of Pressureless Sintering. Ceramics.

[104] Pašti I.A., Jovanović A., Dobrota A.S., Mentus S.V., Johansson B., Skorodumova N.V. (2018). Atomic adsorption on graphene with a single vacancy: Systematic DFT study through the periodic table of elements. Phys. Chem. Chem. Phys..

[105] Sharma R., Baik J.H., Perera C.J., Strano M.S. (2010). Anomalously large reactivity of single graphene layers and edges toward electron transfer chemistries. Nano Lett..

[106] Mu X.N., Chen P.W., Zhang H.M., Cheng X.W., Liu L., Ge Y.X. (2021). Interface-dependent failure behaviors in graphene nanoflakes reinforced Ti matrix composites. Mater. Lett..

[107] Liu J., Jiang W., Liu J., Long D., Wang J. (2020). Atomic-Level Understanding Layer-by-Layer Formation Process of TiCx on Carbon Film. Electrochim. Acta.

[108] Mu X.N., Zhang H.M., Chen P.W., Cheng X.W., Wang B., Liu L., Ge Y.X., Duan H.Q. (2021). Towards high performance GNFs/Ti composite through simultaneously manipulating laminated microstructure and interface reaction. Mater. Sci. Eng. A.

[109] Markovsky P.E., Janiszewski J., Stasyuk O.O., Bondarchuk V.I., Savvakin D.G., Cieplak K., Goran D., Soni P., Prikhodko S.V. (2021). Mechanical Behavior of Titanium Based Metal Matrix Composites Reinforced with TiC or TiB Particles under Quasi-Static and High Strain-Rate Compression. Materials.

[110] Chang S., Du W., Zhao Z., Bai P. (2023). Microstructure and high temperature-mechanical properties of TiC/graphene/Ti6Al4V composite formed by laser powder bed fusion. Metals.

[111] Yan Q., Chen B., Zhang B., Zhang T., Wan J., Shen J., Kou H.C., Li J.S. (2022). Inhibiting the interfacial reaction between few-layered graphene and titanium via SiC nanoparticle decoration. J. Alloys Compd..

[112] Mao Z., Farkoosh A.R., Seidman D.N. (2024). Effects of alloying elements on carbon diffusion in the austenite (f.c.c.) and ferrite (b.c.c.) phases. arXiv.

[113] Taheridoustabad I., Khosravi M., Yaghoubinezhad Y. (2021). Fabrication of GO/RGO/TiC/TiB_2_ nanocomposite coating on Ti–6Al–4V alloy using electrical discharge coating and exploring its tribological properties. Tribol. Int..

[114] Ren W., Zhang W., Zhou S., Zhou Q., Wei J., Wu P., Liu M., Wang X. (2023). Achieving high strength-ductility in TiBw-GNPs/Ti6Al4V composites via 3D interface configuration. J. Alloys Compd..

[115] Huang L.J., Wang S., Dong Y.S., Zhang Y.Z., Pan F., Geng L., Peng H.X. (2012). Tailoring a novel network reinforcement architecture exploiting superior tensile properties of in situ TiBw/Ti composites. Mater. Sci. Eng. A.

[116] Wang S., Huang L., An Q., Jiang S., Zhang R., Geng L., Qu S., Peng H. (2019). Regulating crack propagation in laminated metal matrix composites through architectural control. Compos. Part B Eng..

